# *PPM1D* Mutations Drive Clonal Hematopoiesis in Response to Cytotoxic Chemotherapy

**DOI:** 10.1016/j.stem.2018.10.004

**Published:** 2018-11-01

**Authors:** Joanne I. Hsu, Tajhal Dayaram, Ayala Tovy, Etienne De Braekeleer, Mira Jeong, Feng Wang, Jianhua Zhang, Timothy P. Heffernan, Sonal Gera, Jeffrey J. Kovacs, Joseph R. Marszalek, Christopher Bristow, Yuanqing Yan, Guillermo Garcia-Manero, Hagop Kantarjian, George Vassiliou, P. Andrew Futreal, Lawrence A. Donehower, Koichi Takahashi, Margaret A. Goodell

**Affiliations:** 1 Translational Biology and Molecular Medicine Graduate Program and Medical Scientist Training Program, Baylor College of Medicine, Houston, TX 77030, USA; 2 Department of Pediatrics, Section of Hematology Oncology, Baylor College of Medicine, Houston, TX 77030, USA; 3 Center for Cell and Gene Therapy, Baylor College of Medicine, Houston, TX 77030, USA; 4 Department of Molecular Virology and Microbiology, Baylor College of Medicine, Houston, TX 77030, USA; 5 Haematological Cancer Genetics, Wellcome Sanger Institute, Hinxton, Cambridge CB10 1SA, UK; 6 Wellcome-MRC Stem Cell Institute, Cambridge Biomedical Campus, University of Cambridge, Cambridge CB2 0XY, UK; 7 Department of Genomic Medicine, The University of Texas MD Anderson Cancer Center, Houston, TX 77030, USA; 8 Center for Co-Clinical Trials, The University of Texas MD Anderson Cancer Center, Houston, TX 77030, USA; 9 Department of Neurosurgery, University of Texas Health Science Center at Houston, Houston, TX 77030, USA; 10 Department of Leukemia, The University of Texas MD Anderson Cancer Center, Houston, TX 77030, USA

**Keywords:** clonal hematopoiesis, CHIP, *PPM1D*, t-AML, t-MDS, topoisomerase inhibitors, cisplatin, doxorubicin, etoposide, DNA damage response

## Abstract

Clonal hematopoiesis (CH), in which stem cell clones dominate blood production, becomes increasingly common with age and can presage malignancy development. The conditions that promote ascendancy of particular clones are unclear. We found that mutations in *PPM1D* (protein phosphatase Mn^2+^/Mg^2+^-dependent 1D), a DNA damage response regulator that is frequently mutated in CH, were present in one-fifth of patients with therapy-related acute myeloid leukemia or myelodysplastic syndrome and strongly correlated with cisplatin exposure. Cell lines with hyperactive PPM1D mutations expand to outcompete normal cells after exposure to cytotoxic DNA damaging agents including cisplatin, and this effect was predominantly mediated by increased resistance to apoptosis. Moreover, heterozygous mutant *Ppm1d* hematopoietic cells outcompeted their wild-type counterparts *in vivo* after exposure to cisplatin and doxorubicin, but not during recovery from bone marrow transplantation. These findings establish the clinical relevance of *PPM1D* mutations in CH and the importance of studying mutation-treatment interactions.

**Video Abstract:**

## Highlights

• *PPM1D* is mutated in ∼20% of patients with therapy-related AML or MDS
• *PPM1D* mutations are associated with prior exposure to specific DNA-damaging agents
• Mutant PPM1D confers a survival advantage after cisplatin-induced stress
• *PPM1D* mutants lack an advantage under bone marrow transplantation stress

## Graphical Abstract

**Figure:**
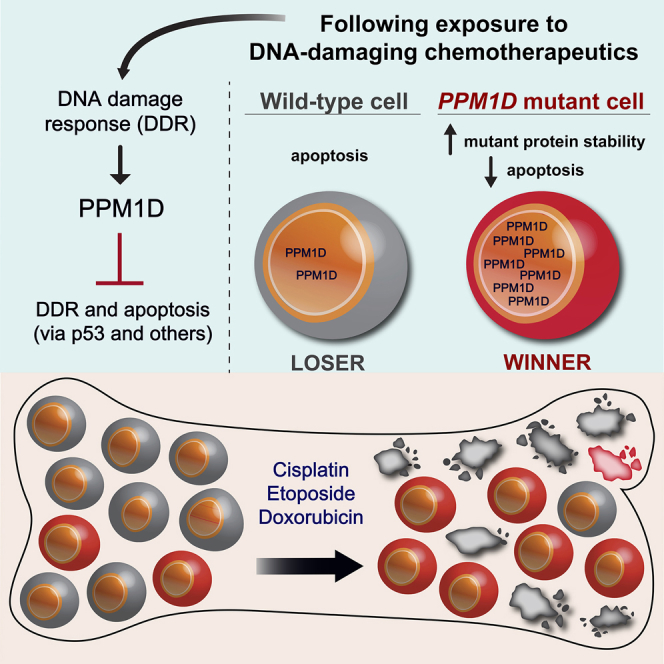

## Teaser

Cytotoxic chemotherapies put patients at risk for future hematopoietic malignancies. Goodell and colleagues show that *PPM1D* mutations confer a survival advantage onto hematopoietic clones by rendering them resistant to DNA-damaging agents such as cisplatin. Selective pressures will be specific to different mutations and should be considered in choice of chemotherapy.

## Introduction

One of the most serious risks of cytotoxic chemotherapies for cancer is the development of secondary, hematopoietic malignancies some years in the future. In theory, chemotherapy and radiation might directly inflict the DNA damage that, when inappropriately repaired, produces the subsequent cancer-driving mutation. It is also possible, however, that cancer therapies might exert selective pressures on hematopoietic stem cells (HSCs) such that certain mutant populations (known as clones) have a selective advantage under cytotoxic conditions. If the mutant clones survive longer, they may accumulate more mutations with time. This could be why the expansion of mutant clones, known as clonal hematopoiesis (CH), is associated with an increased risk of developing hematologic malignancies (Genovese et al., 2014, Jaiswal et al., 2014, Xie et al., 2014): certain mutant clones could serve as premalignant cells with some sort of growth advantage, which then acquire cancer-driving mutations. This is a compelling model. Indeed, deep sequencing before and after chemotherapy has shown that *TP53* mutant cells can pre-exist at low frequencies in the bone marrow prior to chemotherapy and then rise in proportional contribution afterward, likely due to a selective advantage (Wong et al., 2015). Yet, not all CH mutations detected in the blood prior to therapy subsequently evolve into a malignant clone (Berger et al., 2018, Gillis et al., 2017, Takahashi et al., 2017). In fact, CH can be detected in 95% of healthy adults (Young et al., 2016), yet most expanded clones do not evolve into leukemia (reviewed in Bowman et al., 2018). At this point, the nature of the association between CH and malignancy is not clear.

CH has recently been associated with mutations in *PPM1D* (protein phosphatase Mn^2+^/Mg^2+^-dependent 1D), which is part of the DNA damage response pathway. PPM1D is part of a regulatory feedback loop with p53: activated p53 induces expression of PPM1D, which then both directly and indirectly dephosphorylates p53, leading to downregulation of p53-mediated apoptosis (Dudgeon et al., 2013, Lu et al., 2008). *PPM1D* has been found to be amplified and overexpressed in a significant fraction of medulloblastoma, breast cancer, and ovarian cancer (Castellino et al., 2008, Lambros et al., 2010, Tan et al., 2009). Interestingly, truncated forms—the same mutations identified in CH—have been identified in various cancers (The Cancer Genome Atlas Research Network, 2014, Kleiblova et al., 2013, Zajkowicz et al., 2015, Zhang et al., 2014), and these mutations have been observed in patients previously exposed to chemotherapy for solid tumors (Coombs et al., 2017, Gibson et al., 2017, Pharoah et al., 2016, Swisher et al., 2016, Wong et al., 2018). Mutations in *PPM1D* are typically nonsense or frameshift mutations in the sixth exon, which produce a C-terminal truncated protein. Only recently have *PPM1D* mutations been noted in patients with hematologic conditions, specifically therapy-related myelodysplastic syndrome (Lindsley et al., 2017). These findings prompted us to explore the relationship between *PPM1D*, CH, and hematologic malignancies.

Given that *PPM1D* mutations have been associated with CH in patients with prior exposure to cytotoxic therapy (Coombs et al., 2017, Wong et al., 2018), we began our investigation with the therapy-related acute myeloid leukemia (t-AML) and therapy-related myelodysplastic syndrome (t-MDS) that arise in some individuals years after chemotherapy for solid tumors or non-myeloid hematologic malignancies.

## Results

### PPM1D Mutations Are Relatively Common in Therapy-Related AML and MDS

**Figure 1:**
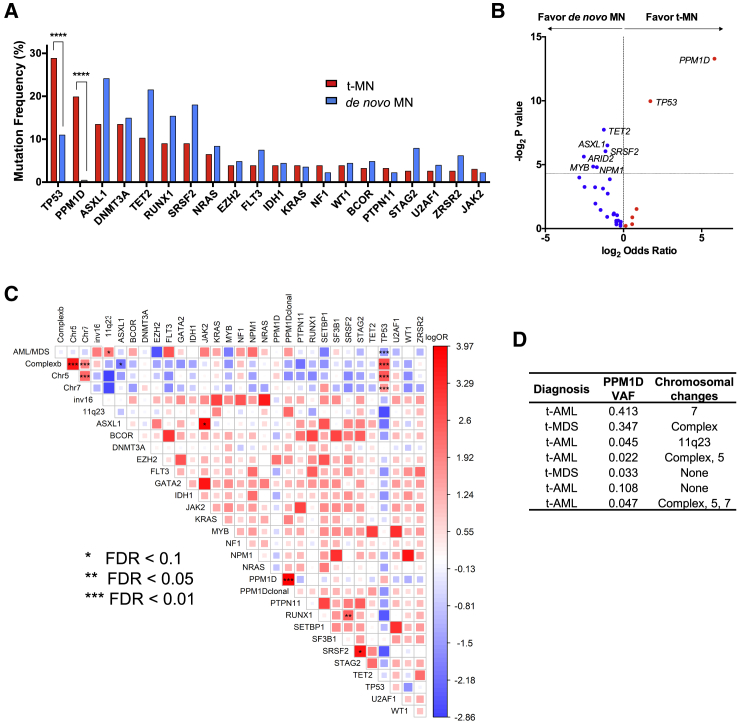
Mutational Landscape of Myeloid Neoplasm (MN)-Associated Genes in the t-AML/t-MDS Cohort (A) The twenty most frequently mutated genes detected by targeted gene sequencing in the t-AML/t-MDS study cohort (n = 156) are shown. The red bars represent the mutation frequency in the t-MN (t-AML/t-MDS) cohort and the blue bars represent the mutation frequency in a matched *de novo* MN (AML/MDS) control cohort (n = 228). (B) Volcano plot of genes enriched in t-AML/t-MDS compared to *de novo* AML/MDS. The horizontal dotted line corresponds to a p value of 0.05. (C) Pairwise association plot of overall mutation co-occurrence or mutual exclusivity, adjusted for multiple comparisons. Blue represents a negative association (mutual exclusivity) while red represents a positive association (co-occurrence). The magnitude of association is represented by both the size of the square and color gradient, which corresponds to a range of log odds ratio values. The statistical significance of associations is represented by the false discovery rate (FDR). The asterisks indicate the level of significance (FDR 0.1, 0.5, and 0.01). “PPM1D clonal” refers to the subset of *PPM1D* mutated cases with VAF > 0.2. (D) Seven cases where *PPM1D* was the only detected somatic mutation out of the 295 sequenced genes. See also Figure S1 and Tables S1 and S2.

We performed targeted-capture sequencing of 295 cancer genes combined with amplicon sequencing on diagnostic bone marrow samples from 156 patients with t-MDS (n = 79) or t-AML (n = 77) (Table S1). *PPM1D* mutations were found in 20% of these cases (31/156) and at similar frequencies in both groups (t-AML: 15/77, 19.5%; t-MDS 16/79, 20.2%). Only *TP53* mutations appeared more frequently (45/156, 28.8%). In contrast, *PPM1D* was mutated in only 1 out of 228 patients in a matched *de novo* AML/MDS cohort (AML n = 121 and MDS n = 107, Table S2), confirming that *PPM1D* mutations are enriched in t-AML/t-MDS arising from prior therapy (odds ratio, 56; 95% confidence interval [CI], 7.6–417.3; p = 0.0001) (Figures 1A and 1B).

**Figure 2:**
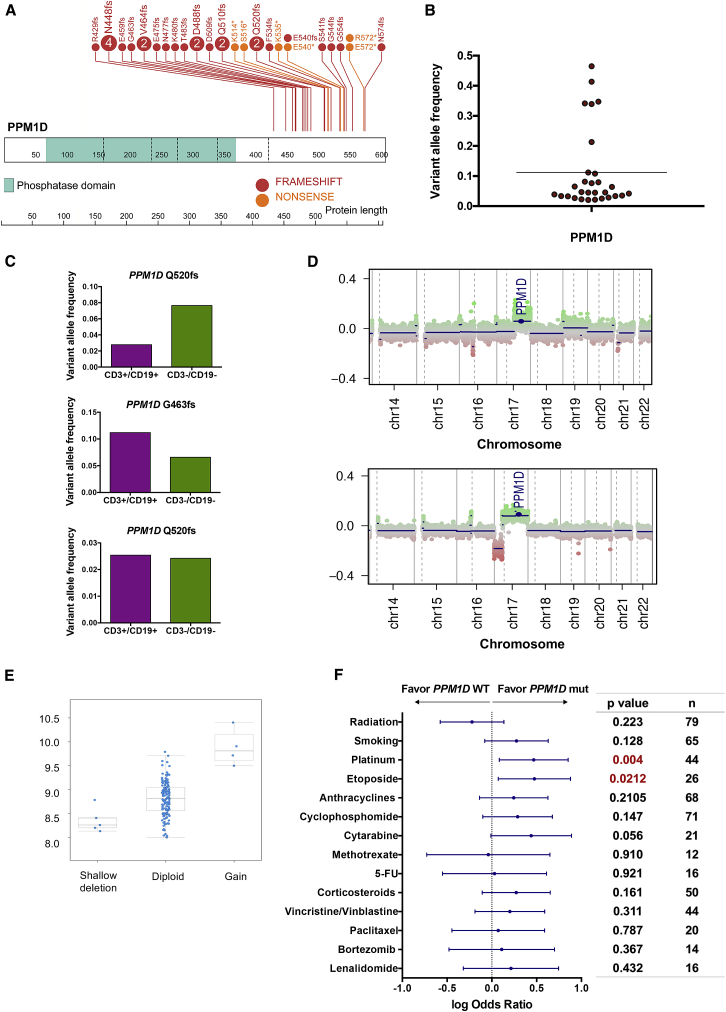
Features of *PPM1D* Mutated t-AML/t-MDS Cases (A) Lollipop plot showing the distribution of *PPM1D* truncating mutations across the final exon of the gene. Vertical dotted lines demarcate the coding exons of the gene, with the corresponding amino acids shown below. The phosphatase domain of the protein is denoted by the green segment. Frameshift mutations are depicted in red and nonsense mutations in orange. The number of patients with each mutation is indicated in the lollipops (circles without numbers represent one case). (B) Variant allele frequency distribution plot in *PPM1D* mutated cases (n = 31; range, 0.02–0.47; mean, 0.1). (C) In three cases, next-generation sequencing was performed to determine the variant allele frequency of *PPM1D* in lymphoid (CD3^+^/CD19^+^) and non-lymphoid (CD3^−^/CD19^−^) peripheral blood fractions. (D) Genome-wide copy number plots of two separate cases with *PPM1D* copy number gain. Chromosome position is shown on the x axis and copy number log2 ratio is shown on the y axis. (E) Copy number alterations of *PPM1D* in 162 *de novo* AML cases from (TCGA, 2013), plotted with the corresponding *PPM1D* mRNA expression level. (F) Forest plot showing the association of *PPM1D* mutations with prior exposure to specific genotoxic agents, per clinical chart review. Log odds ratio is depicted with the 95% confidence interval. Agents with favorable associations with *PPM1D* mutations trend to the right of the dotted line. These include cisplatin (p = 0.004 and FDR = 0.056) and etoposide (p = 0.02 and FDR = 0.148). The p value and total number of patients exposed to each agent are noted to the right. See also Table S3.

Unlike *TP53*, *PPM1D* was not significantly associated with complex cytogenetics or deletions in chromosomes 5 or 7 (Figure 1C) (Christiansen et al., 2001, Godley and Larson, 2008, Lindsley et al., 2017). While the cohort had typical t-AML/t-MDS-associated genetic alterations, we did not observe significant co-mutation or mutual exclusivity of *PPM1D* with other genes nor impact on overall survival (Figures 1C, S1A, and S1B). Notably, *PPM1D* was the sole detected somatic mutation from the panel in seven of the 31 cases, two of which had no detectable co-occurring chromosomal alterations (Figure 1D). *PPM1D* exon 6 mutations in our t-AML/t-MDS cohort were all truncating mutations with no particular hotspot, consistent with the distribution of exon 6 mutations found in CH and solid tumors (Figure 2A) (Dudgeon et al., 2013, Genovese et al., 2014, Ruark et al., 2013, Tan et al., 2009). The variant allele frequency (VAF) of these mutations ranged from 0.02 to 0.47, with a mean of 0.11 and median of 0.05 (Figure 2B). In three patients with *PPM1D* mutations for whom different lineage fractions were available, we detected the mutation in both lymphoid (CD3^+^ CD19^+^) and non-lymphoid (CD3^−^ CD19^−^) fractions, consistent with an HSC or progenitor origin for the *PPM1D* mutation (Figure 2C). We next established a patient-derived xenograft (PDX) model using one of the *PPM1D* mutated t-AML samples and observed that the engrafted leukemic cells indeed carried a clonal *PPM1D* mutation (VAF >0.4). This provides evidence that at least in select cases, *PPM1D* mutant cells can constitute a significant portion of the leukemic clone (Figure S1C).

We also screened for copy number alterations in *PPM1D* with a genome-wide SNP array in the same t-AML/t-MDS cohort (n = 120, as DNA was insufficient for 36 patients). We found two cases (1.7%) with *PPM1D* copy number gain (both t-AML, Figure 2D) and no co-occurring *PPM1D* mutations. Copy number gain of *PPM1D* was also found in 2.4% of *de novo* AML cases analyzed as part of The Cancer Genome Atlas (TCGA) project (TCGA, 2013), (four out of 162 cases, Figure 2E). *PPM1D* copy number gain thus appears to be rare but detectable in both therapy-related and *de novo* AML/MDS and should be screened for along with *PPM1D* mutations to fully characterize *PPM1D* alterations in hematologic malignancies.

### PPM1D Mutations Are Associated with Prior Exposure to Specific DNA-Damaging Agents

To determine whether specific conditions are associated with expansion of PPM1D mutant clones, we reviewed clinical charts for patient exposures (available for 140 of the 156 patients; 25 *PPM1D* and 115 non-*PPM1D* mutated cases). We identified a significant association between prior exposure to platinum agents (cisplatin, carboplatin, and oxaliplatin) and *PPM1D*-mutated t-AML/t-MDS (odds ratio, 2.9; 95% CI, 1.2–7.1; p = 0.004; false discovery rate [FDR] = 0.056). *PPM1D* mutations were also associated with prior exposure to the topoisomerase inhibitor, etoposide (odds ratio, 2.98; 95% CI, 1.2–7.6; p = 0.02; FDR = 0.148) (Figure 2F). There was no association between *PPM1D* mutations and prior radiation therapy or smoking (p = 0.223 and 0.128, respectively), the latter of which is consistent with prior observations (Coombs et al., 2017). We also found no significant association between *PPM1D* mutations and the nature of the primary malignancy (Table S3).

**Figure 3:**
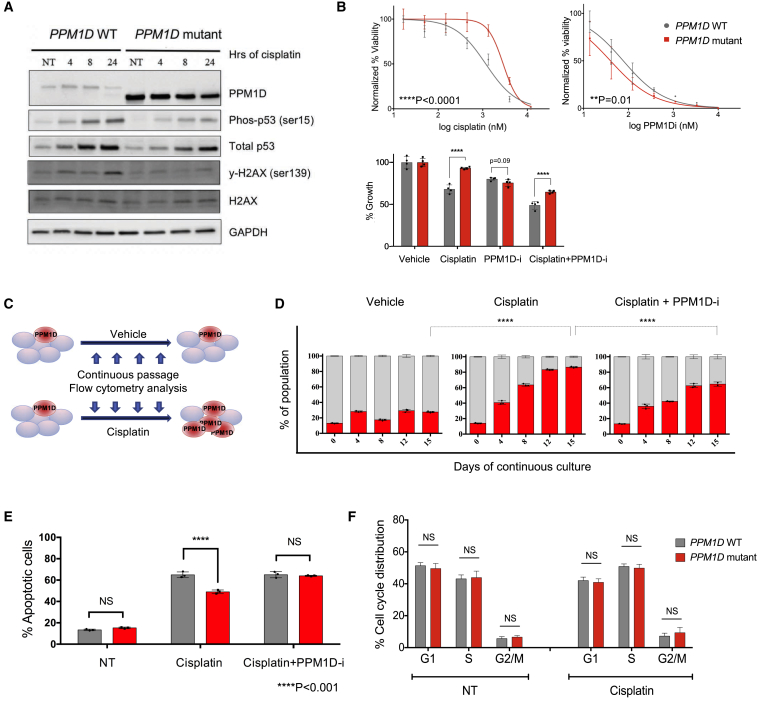
*PPM1D* Mutants Resist Cisplatin-Induced Apoptosis (A) Immunoblot of *PPM1D* WT and mutant HEK293 cells in the presence and absence of DNA damage. Cells were treated with 30 μM cisplatin, harvested at 4, 8, and 24 hr, and probed with the indicated antibodies. A composite of images is shown (see STAR Methods for details). (B) Top: dose-response curves for cell viability with cisplatin and a specific PPM1D inhibitor (GSK2830371) in WT and *PPM1D* mutant MOLM13 lines. Mean ± SD (n = 3) is shown along with a non-linear regression curve. All values are normalized to the baseline cell viability with vehicle, as measured by the WST-8 assay. The IC_50_ of cisplatin was 1.2 μM and 2.8 μM for the *PPM1D* WT and mutant lines, respectively (p < 0.001). Bottom: cell viability measured with WST-8 under combination treatment with cisplatin (1 μM) and GSK2730371 (a PPM1D inhibitor; 250 nM). (C) Schematic of experimental strategy shown in (D). GFP-negative *PPM1D* mutant cells were mixed with GFP-positive control cells at a starting ratio of 20:80 and subjected to treatment with vehicle (water) or cisplatin (+/− 18 nM GSK2830371). Population dynamics were assessed by flow cytometry every 4 days for 15 days. (D) Each bar depicts the proportion of *PPM1D* WT cells (in gray) and mutant cells (in red) in culture, measured at the indicated time points. Data represent mean ± SD of triplicates (n = 3). At least three independent experiments were conducted for each experiment shown above, with similar findings. The corresponding flow cytometry plots are depicted in Figure S3A. (E) *PPM1D* mutations confer resistance to cisplatin-induced apoptosis. *PPM1D* WT and mutant cells were treated with 1 μM cisplatin (+/− 24nM GSK2830371) for 72 hr, incubated with annexin V-APC and 7-AAD, and analyzed using flow cytometry. The percentage of annexin V positive (late and early apoptotic) cells is represented in the histogram (mean +/− SD shown). The experiment was performed in triplicate (n = 3). (F) *PPM1D* WT and mutant cells were treated with 750 nM cisplatin (or vehicle) for 24 hr and fixed for BrdU cell-cycle analysis. Anti-BrdU FITC antibody and propidium iodide (PI) were used to distinguish cells with active synthesis and DNA content, respectively. Mean values and SD are shown (n = 6). Three independent experiments were performed with similar findings. See also Figures S2, S3, and S4.

To explore how platinum agents might confer selective advantage on *PPM1D* mutant cells, we generated isogenic *PPM1D* mutant and wild-type (WT) human cell lines using CRISPR-Cas9 in both MOLM13 and HEK293 cells (Figures S2A and S2E). We first needed to confirm that cisplatin treatment would activate the DNA damage response and induce PPM1D protein expression in our cell lines, because cisplatin interferes with DNA replication via DNA adduct formation. In unedited cells, PPM1D levels increased, along with phospho-p53 and γ-H2AX. In the *PPM1D* mutant cells, PPM1D increased by 16-fold even in the absence of DNA damage (Figure S2B), strongly suppressing phospho-p53 and γ-H2AX (Figure 3A). As expected, the elevated protein levels reflect the increased stability of the truncated protein, rather than changes in the intrinsic phosphatase activity of PPM1D, as demonstrated by treatment with proteasome inhibitors and an *in vitro* phosphatase assay (Figures S2C and S2D) (Kleiblova et al., 2013).

We next performed dose-response experiments with our isogenic *PPM1D* WT and mutant cell lines, testing their relative survival following exposure to cisplatin and other chemotherapeutic agents. We performed these experiments with CRISPR-generated isogenic clones derived from three different leukemic cell lines (MOLM13, OCI-AML2, and OCI-AML3), with technical triplicates for each. The *PPM1D* truncating mutation conferred significant chemoresistance to cisplatin (p < 0.0001) (Figure 3B). We then tested whether a specific PPM1D inhibitor, GSK2830371, which targets both the phosphatase activity and degradation of PPM1D (Gilmartin et al., 2014), could render the cells more sensitive to cisplatin. *PPM1D* mutant cells were more sensitive to GSK2830371 than WT cells, and combination treatment with cisplatin and GSK2830371 sensitized the mutants to cisplatin (Figure 3B).

### PPM1D Mutations Confer Resistance to Apoptosis

To better understand the relative fitness conferred by *PPM1D* mutation, we studied mixed populations of cells in culture at a ratio of 80:20 (GFP-positive WT to GFP-negative mutant *PPM1D* cells). We tracked population growth by flow cytometry, with and without continuous cisplatin treatment (Figures 3C and S3A–S3C). In the absence of cisplatin, the mutant cells exhibited a minor growth advantage. With cisplatin, however, the mutant cells expanded from 20% to over 80% of the population over 15 days. Concurrent administration of cisplatin and the PPM1D inhibitor GSK2830371 attenuated the growth of the *PPM1D* mutants, confirming that high expression of PPM1D confers the selective advantage (Figure 3D). An independent experiment revealed similar findings (Figure S3B), as did an additional experiment with reciprocally labeled cells (GFP-positive mutant *PPM1D* cells mixed with GFP-negative WT cells), which we performed to confirm that the advantage of the mutants was not due to silencing of the GFP transgene. Consistent with earlier findings, GFP-positive *PPM1D* mutants expanded from 10% to 45% over 15 days of cisplatin treatment (Figure S3C). Together, these findings clearly demonstrate that *PPM1D* mutations confer a selective advantage in the context of cisplatin.

Selection for the mutant cells could occur via multiple cellular mechanisms. Given that PPM1D mutants strongly suppress p53, we hypothesized that the mutant cells would be resistant to cisplatin-induced apoptosis. This appeared to be the case: following one dose of cisplatin, we observed 49% apoptotic mutant cells compared to 65% apoptotic WT cells. The PPM1D inhibitor GSK2830371 restored normal levels of apoptosis to the mutant cells (Figures 3E and S4A).

An altered proliferation rate by *PPM1D* mutant cells could also contribute to a fitness advantage. To examine this possibility, we performed bromodeoxyuridine (BrdU) cell-cycle analysis in isogenic WT and *PPM1D* mutant cells (MOLM13) at baseline and after 24 hr of 750 nM cisplatin treatment. The results showed that prior to cisplatin treatment, there were no differences between WT and mutant cells (Figures 3F and S4B). Importantly, cisplatin treatment induced cell-cycle arrest in both mutant and WT cells, as evidenced by accumulation of cells in S phase and G2/M phase. However, the proportions of cells in these phases were not different between *PPM1D* mutants and WT cells, indicating that they were similarly arrested with no significant proliferation differences. While we cannot exclude some advantage contributed by proliferation differences that could be revealed under different conditions, our data strongly suggest that resistance to apoptosis is likely the primary contributor to the competitive outgrowth of *PPM1D* mutants.

### PPM1D Mutations Confer Chemoresistance to Specific Agents

**Figure 4:**
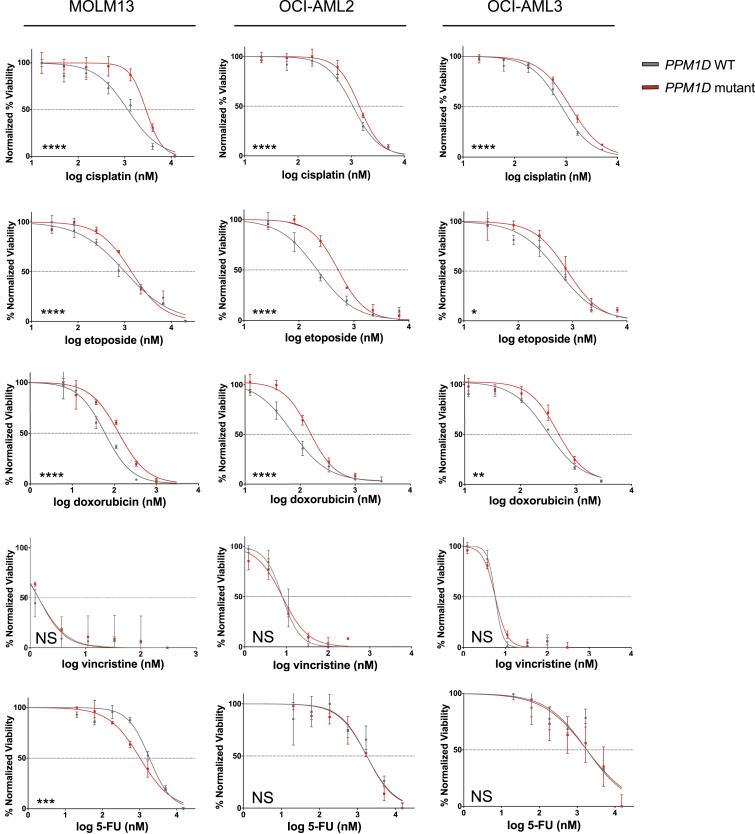
Screen for Chemoresistance in *PPM1D* Mutants Various classes of chemotherapy agents were screened in the isogenic CRISPR-generated WT and *PPM1D* mutant cell lines (MOLM13, OCI-AML2, and OCI-AML3). The chemotherapy agents selected include key agents utilized in the treatment of primary tumors that t-AML/t-MDS patients in our cohort were previously exposed to. Dose response curves are shown, with red representing *PPM1D* mutants and gray representing WT. The data points represent mean ± SD of triplicates (n = 3).

To extend the characterization of *PPM1D* mutant chemoresistance, we first assessed the relative sensitivity of *PPM1D* mutant and WT cells to additional chemotherapeutics with distinct mechanisms of action. We performed dose-response assays in isogenic *PPM1D* WT and mutant lines across three different leukemic cell lines. Compared to WT cells, *PPM1D* mutants showed greater resistance to doxorubicin and etoposide, the latter of which is consistent with the clinical findings mentioned above (Figure 4). In contrast, *PPM1D* mutants showed no significant differences in sensitivity to vincristine or to 5-FU relative to WT cells.

**Figure 5:**
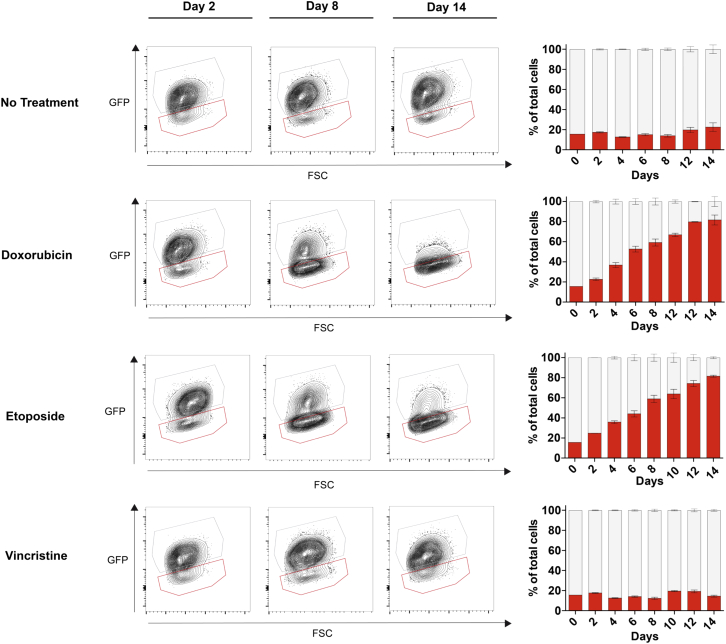
*PPM1D* Mutants Demonstrate a Selective Advantage *In Vitro* with Certain Classes of Chemotherapeutics *In vitro* competition was performed with GFP-negative *PPM1D* mutant cells to GFP-positive control cells at a starting ratio of 15:85. The competing cells were treated with doxorubicin (20 nM), etoposide (250 nM), or vincristine (1 nM) every 4 days, and flow cytometry was performed every 2 days over 14 days to assess the change in percentage of *PPM1D* mutants. Representative flow plots are depicted at three time points: day 2, day 8, and day 14. The red gate denotes the GFP-negative *PPM1D* mutant population, and the gray gate denotes the GFP-positive control population. Mean ± SD are shown in the summary graphs (n = 3).

To determine whether the drug sensitivity profiles translated to a competitive advantage, we modeled cell competition *in vitro* with *PPM1D* mutant (GFP^–^) and WT (GFP^+^) MOLM13 cells, with multiple chemotherapy exposures over 14 days, designed to mimic treatment cycles received by patients. In the context of doxorubicin and etoposide, GFP^–^ cells went from an average proportion below 20% to around 80% over the treatment period, while vincristine provided no selective advantage (Figure 5). These data clearly indicate that *PPM1D* mutants are positively selected with some, but not all classes of agents.

### PPM1D Mutations Confer a Selective Advantage *In Vivo* under Cisplatin Exposure

**Figure 6:**
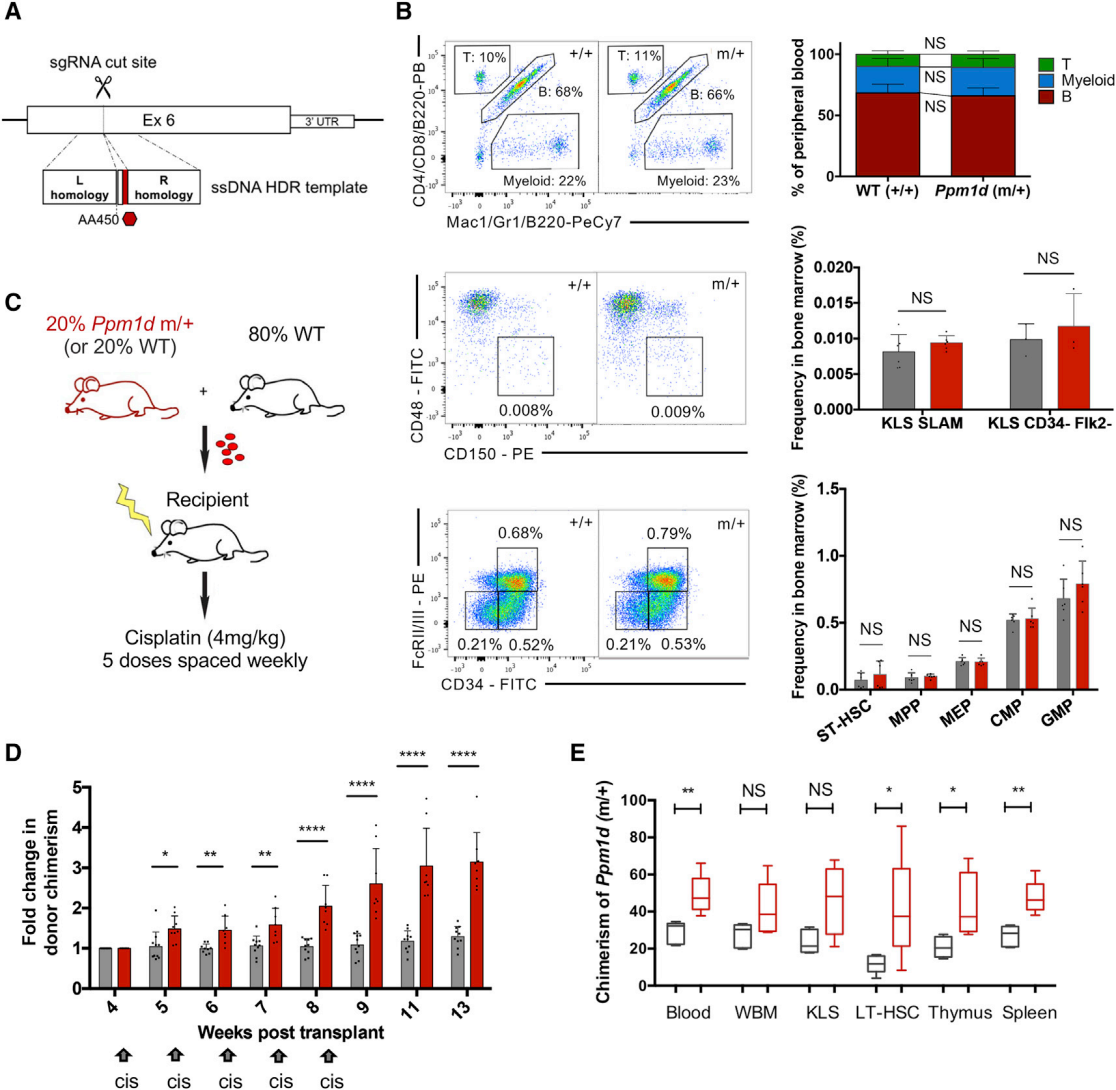
Cisplatin Treatment Confers a Survival Advantage on *Ppm1d-*Mutant Hematopoietic Cells *In Vivo* (A) Generation of the R451X knockin mouse model utilizing CRISPR-Cas9 and homology directed repair. The founder mouse was crossed with WT mice for the F1 generation, and a heterozygous line was subsequently maintained. (B) Representative flow plots showing baseline hematopoietic characterization of the R451X mutant mouse (*Ppm1d 1*^m/+^) compared to WT (*Ppm1d*^+/+^). We assessed lineage composition of the peripheral blood and examined the frequency of different progenitor compartments in the bone marrow by flow cytometry (n = 10 mice/group for peripheral blood, n = 6 mice/group for bone marrow). Long-term hematopoietic stem cells (LT-HSCs) were identified by c-kit^+^ lineage^−^ Sca-1^+^ (KLS), with either CD150^+^ CD48^−^ (“SLAM”) gating (n = 6 mice) or CD34^−^ Flk2^−^ gating (n = 3 mice). Mean +/− SD is shown. (C) Competitive whole bone marrow transplant scheme: 20% of either R451X (*Ppm1d*^m/+^) or control (*Ppm1d*^+/+^) CD45.1/45.2 bone marrow cells were mixed with 80% WT CD45.1 bone marrow cells. A total of 3 × 10^6^ whole bone marrow cells were transplanted into lethally irradiated 8-week-old recipient mice (n = 8 per group). Engraftment was assessed 4 weeks following transplant, and the recipient mice were treated with weekly doses of 4 mg/kg cisplatin (intraperitoneally [i.p.]) for 5 consecutive weeks. (D) Average peripheral blood chimerism 4 weeks following transplant was 22.8% (range 18.3%–27%) in the control cohort (*Ppm1d*^+/+^) and 13.9% (range 8.2%–20.4%) in the mutant cohort (*Ppm1d*^m/+^). Chimerism was monitored weekly by flow cytometry. The graph depicts the average of the fold change in chimerism for each mouse relative to the initial chimerism at 4 weeks post-transplant (n = 8 for each group, mean ± SD shown). ^∗^p < 0.05, ^∗∗^p < 0.01, ^∗∗∗^p < 0.001, and ^∗∗∗∗^p < 0.0001. (E) Bone marrow was harvested from the *Ppm1d*^m/+^ competitively transplanted mice to assess chimerism on multiple levels of the hematopoietic hierarchy, including LT-HSC, KLS, and whole bone marrow (WBM) cells (cisplatin-treated in red and non-treated in gray, n = 5 mice per group). Data are represented by box-and-whisker plots, with the quartiles, minimum, and maximum values shown. See also Figures S5, S6, and S7.

To examine the effect of truncated PPM1D on hematopoiesis and the variables that drive *PPM1D-*mutant clonal expansion *in vivo*, we generated a mouse model with a *Ppm1d* truncation at R451 (R451X), which is equivalent to the R458X human *PPM1D* mutation commonly found in CH (Genovese et al., 2014) (Figures 6A and S5A). The heterozygous *Ppm1d* R451X mice (*Ppm1d*^m/+^) were viable and fertile, with no apparent phenotypic abnormalities. We generated primary mouse embryonic fibroblasts (MEFs) from *Ppm1d* mutant and WT littermates and confirmed abundant truncated protein and dephosphorylated p53 even in the absence of DNA damage (Figure S5B). R451X mutant MEFs also demonstrated resistance to apoptosis upon cisplatin treatment (Figure S5C). Because *PPM1D* mutations are consistently found in a heterozygous state in both CH and t-AML/MDS patients, we used the heterozygous mice for downstream hematopoietic studies.

Previous work suggests that *PPM1D* mutations arise in hematopoietic stem cells rather than later myeloid and lymphoid cells (Wong et al., 2018), so we began by analyzing the lineage composition of the peripheral blood in 8-week-old *Ppm1d* mutant and WT mice. There were no appreciable differences in proportions of long-term or short-term HSCs, multipotent progenitors, megakaryocyte erythrocyte progenitors, or common myeloid progenitors (Figures 5B and S6A–S6C).

To better understand the parameters of *PPM1D* mutant cell fitness, we performed whole bone marrow transplantation with the donor marrow consisting of 20% *Ppm1d*^m/+^ (mutant) or 20% *Ppm1d*^+/+^ (control) cells mixed with 80% WT cells (n = 8 per group) (Figure 6C), using CD45 allelic differences to track the test and WT cell populations. Following engraftment at 4 weeks, cisplatin was administered over 5 cycles via weekly intraperitoneal injection to mimic the cycles of chemotherapy received by patients for their primary cancers. When control cells were mixed with WT cells in a 20/80 ratio and exposed to cisplatin, we observed minimal changes in peripheral blood chimerism over time (Figure 6D). In contrast, when 20% *Ppm1d* R451X cells were mixed with 80% WT cells, the mutants outgrew the WT cells as early as 1 week after the first cisplatin injection. Even after cisplatin treatment was stopped 8 weeks post-transplantation, the mutant cells continued to outgrow the WT, from an average blood contribution of 13.9% (range of 8.2%–20.4%) at 4 weeks to an average of 42% (range of 31.0%–59.8%) at 13 weeks (Figures 6D and S7A). (Even though the bone marrow transplant contained 20% mutant cells, apparently fewer than 20% of the mutant progenitors/HSCs successfully engrafted in the recipient mice. Thus, their initial contribution measured at 4 weeks is <20%.) Analysis of the bone marrow of recipients 16 weeks after transplantation revealed that the contribution of *Ppm1d-*mutant cells increased at every level of the hematopoietic hierarchy, from long-term (LT-) HSCs to mature cells, following cisplatin treatment (Figure 6E). We validated these results in a repeat transplant (n = 15), after which we observed two waves of *Ppm1d* mutant expansion spaced by 8 weeks (Figure S7B). These waves suggest an immediate survival advantage for mature *Ppm1d* mutant blood cells followed by the emergence of differentiated progeny derived from mutant stem and progenitor cells that were selected for during cisplatin treatment.

As our *in vitro* data suggested that *PPM1D* mutations confer an advantage in the context of some drugs, but not others, we further examined the competitive cellular dynamics with additional agents in our mouse model. Utilizing the same strategy described above (20% *Ppm1d* mutant or WT cells mixed with 80% WT cell in a bone marrow transplant), we treated mice with doxorubicin or vincristine following previously published treatment regimens for mice (Chao et al., 2015, Zuber et al., 2009) starting 8 weeks after transplant. Over three rounds of doxorubicin administration (n = 8 per group), we observed a significant difference in average *Ppm1d* mutant chimerism at the final time point, compared to that of WT cells in the control transplant (36.5% versus 16.0%, respectively, p = 0.005). In contrast, after eight weekly administrations of vincristine (n = 5 per group), *Ppm1d* mutant chimerism was not significantly different from that of WT cells (15.4% versus 20.5%, respectively, p = 0.37) (Figure S7C). Together, these data establish that *Ppm1d* mutations confer a selective advantage on hematopoietic cells *in vivo* in the context of some chemotherapeutic agents but not others.

### Not All Stressors Favor Expansion of PPM1D Mutant Clones

**Figure 7:**
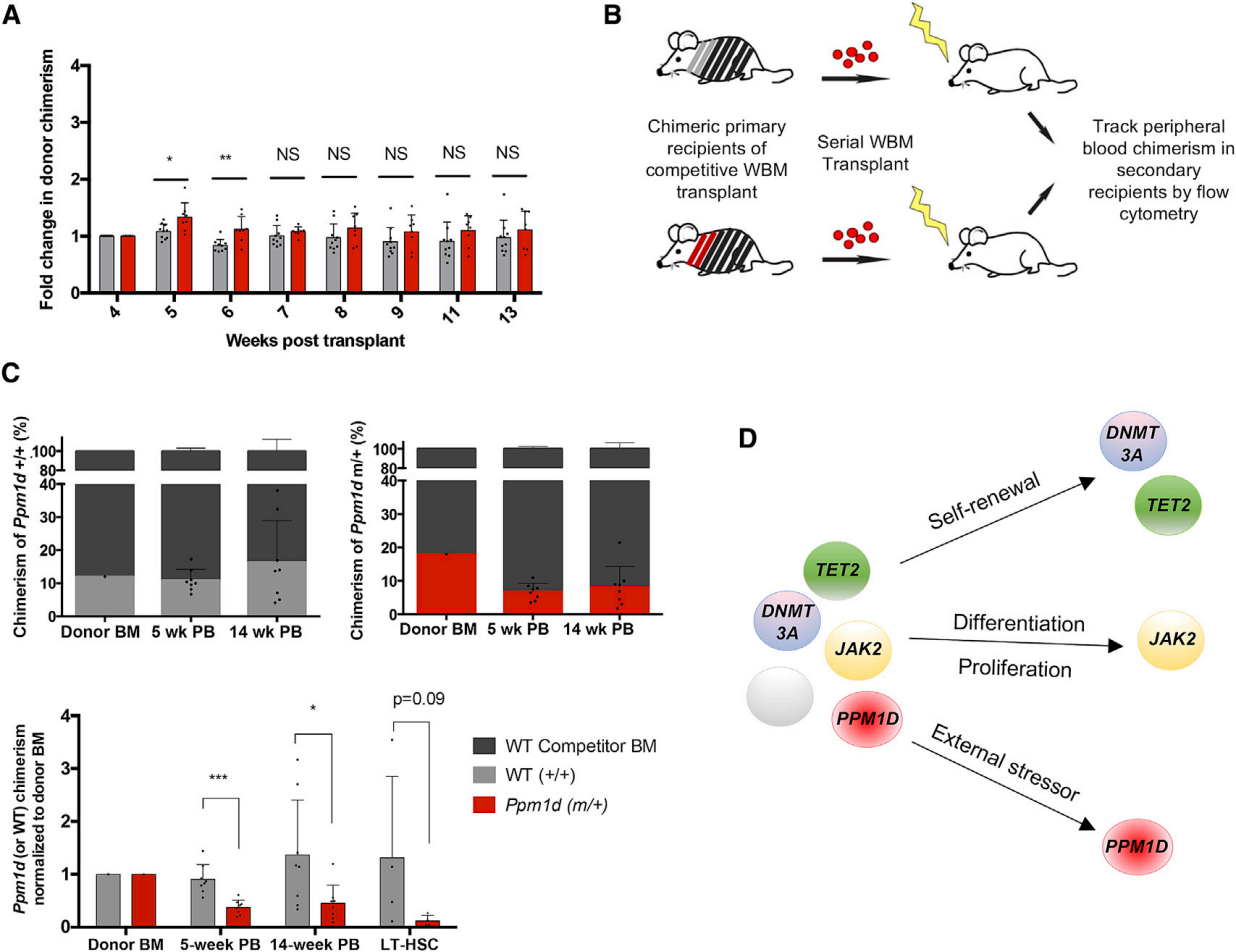
*Ppm1d* Mutant Cells Lose Their Survival Advantage in the Context of Bone Marrow Transplantation (A) Competitive whole bone marrow transplant was performed, with 20% of either R451X (*Ppm1d*^m/+^) or control (*Ppm1d*^+/+^) bone marrow cells mixed with 80% WT bone marrow cells. The recipient mice (n = 8 per group) were not treated with chemotherapy following transplant. Chimerism was monitored weekly by flow cytometry. Normalized values are shown using the initial chimerism at 4 weeks as the baseline for calculation of fold change (mean ± SD shown). ^∗^p < 0.05, ^∗∗^p < 0.01, ^∗∗∗^p < 0.001, and ^∗∗∗∗^p < 0.0001. (B) Schematic of the serial bone marrow transplantation performed 13 weeks after the initial competitive transplant shown in (A). 3 × 10^6^ whole bone marrow (WBM) cells from the primary recipients of the transplant were serially transplanted into lethally irradiated secondary recipient mice (n = 8 per group). Peripheral blood chimerism was assessed in the secondary recipients 5 and 14 weeks following serial transplantation. (C) Graphs depicting the chimerism of the donor bone marrow (BM) at the time of serial transplantation, the chimerism in the peripheral blood (PB) of the secondary recipients at 5 and 14 weeks after transplantation (n = 8 per group), and the chimerism in LT-HSCs (n = 4 per group) at 18 weeks. The summary bar graph below shows chimerism at each time-point, normalized to the initial chimerism of transplanted bone marrow (red, *Ppm1d* mutant; gray, WT control). Mean ± SD is shown. (D) Model of clonal hematopoiesis, emphasizing that different genes have different fitness effects in different contexts. Intrinsic factors such as self-renewal have been shown to drive clonal expansion of *DNMT3A* and *TET2* mutants, whereas aberrant differentiation and proliferation drive expansion of *JAK2* mutations. With *PPM1D*, extrinsic stressors such as cisplatin promote expansion of the mutants. See also Figure S7.

To determine whether *Ppm1d* mutant clones resist cellular stress in general, we tested their response to the severe stress of serial bone marrow transplantation, which requires multiple rounds of rapid HSC expansion (Sun et al., 2014). We established a transplant cohort with donor whole marrow consisting of 20% *Ppm1d-*mutant or control cells mixed with 80% WT cells, and monitored peripheral blood chimerism longitudinally following transplant, in the absence of chemotherapy. There was no significant expansion of either the control cells or *Ppm1d* mutant cells over 13 weeks, as determined from the peripheral blood chimerism (Figures 7A and S7A). We then performed serial transplantation with whole bone marrow from the primary transplanted mice (n = 8 recipients for each group, 3 × 10^6^ whole bone marrow cells transplanted into each recipient mouse) (Figure 7B). *Ppm1d* mutant cells engrafted and reconstituted the peripheral blood less effectively than WT cells, as reflected in their chimerism at 5 weeks and 14 weeks after serial transplantation (Figure 7C). At 20 weeks, the average LT-HSC chimerism was lower with *Ppm1d* mutants than with WT (2.5% versus 15.8%; n = 4; p = 0.19), confirming that *Ppm1d* mutant LT-HSCs lack a self-renewal advantage (Figure S7D). A tertiary serial transplantation revealed similar findings, where *Ppm1d* mutant cells demonstrated significantly lower contribution to the peripheral blood than WT cells, even when normalized to donor bone marrow chimerism (Figure S7D).

These results suggest that the stress of hematopoietic transplantation and HSC engraftment has a neutral, and perhaps negative, impact on selection for *Ppm1d* mutants. Bone marrow transplantation imposes distinct stress on HSCs compared to cytotoxic agents, and we cannot distinguish what aspects of HSC function are impaired; possibly the engraftment capacity of *Ppm1d* mutant cells is reduced. This is suggested by the defect seen as early as 5 weeks after transplantation. These data are consistent with clinical findings showing a decrease in the variant allele frequency (VAF) of *PPM1D-*mutant clones following autologous transplantation in patients (Wong et al., 2018). Interestingly, the poor engraftment capability of *Ppm1d* mutant HSCs has some parallels with phenotypes seen in *Ppm1d* knockout (KO) HSCs (Chen et al., 2015). In these KO mice however, the reconstitution defect is attributed to defects in HSC differentiation. Clearly, *Ppm1d* loss (as in the KO mice) versus hyperactivity (as in the CH-associated mutations) have very distinct effects on HSC function, and suggest that WT PPM1D levels may be optimal for HSC homeostasis.

## Discussion

Cancer therapies create a variety of conditions that favor the death of malignant cells. Necessarily, these various conditions will also favor the survival of other cells —usually not WT. Pre-existing somatic mutations may contribute to the outcome for individual stem cells. While many somatic mutations will have a neutral effect, others may confer upon an HSC clone a greater fitness through enhanced self-renewal capacity, an augmented rate of proliferation, or a diminution in susceptibility to cell death. Expansion of HSCs with mutations in *TET2* and *DNMT3A*, for example, appears to be driven by increased self-renewal (Challen et al., 2011, Kunimoto et al., 2012, Moran-Crusio et al., 2011). Other genes such as *JAK2* skew normal proliferation and differentiation of hematopoietic progenitor cells, resulting in strong expansion (James et al., 2005). The mechanism of expansion of cells with *PPM1D* mutations is unusual in that its selective advantage arises specifically in the context of chemotherapy with agents that induce apoptosis (Figure 7D).

In the context of cisplatin, our results demonstrate that resistance to apoptosis plays a significant role in the competitive advantage of *PPM1D* mutant cells. Even though their resistance to apoptosis seems modest initially (49% apoptosis in mutant versus 65% in WT cells) (Figure 3), after multiple rounds of exposure, this ∼16% difference is compounded. For example, after one exposure to cisplatin, the ratio of live mutant:WT cells is 51:35 (1.46). Over four rounds of treatment, with a starting proportion of *PPM1D* mutant cells at 20% and assuming no proliferation differences, the theoretical ratio of mutant:WT cells would go to 90% (20%^∗^1.46ˆ4). This is remarkably close to our observations (85% mutant cells in Figure 3 and 45% mutant cells from a starting portion of 10% in Figure S3C).

A competitive advantage could also be conferred by diminished cell-cycle arrest after chemotherapeutic treatment. *PPM1D-*mutant and WT cells both appeared to experience cell-cycle arrest to a similar extent following cisplatin treatment, again suggesting that differences in response to apoptosis may be the primary mechanism through which *PPM1D* mutant cells predominate. This is consistent with prior observations in ovarian cancer cells, where the PPM1D mutant protein was found to play a role in cisplatin resistance via attenuation of p53-dependent apoptosis (Ali et al., 2012). However, we cannot exclude some effect of *PPM1D* mutations on cellular proliferation in some contexts, as recently suggested for cytarabine treatment (Kahn et al., 2018). Furthermore, the starting proportion of *Ppm1d* mutant cells in the donor bone marrow prior to chemotherapy exposure could also affect the potency of selection as previously seen with *Tp53* mutant cells in mice (Bondar and Medzhitov, 2010). Taken together, our findings suggest that *PPM1D* mutant clones are present in a small proportion of the HSC population prior to chemotherapy. Cytotoxic drug treatments then create a fitness landscape in which *PPM1D* mutant cells are better adapted for survival than WT cells.

This scenario is similar to what has been shown with *TP53* in the context of chemotherapy (Wong et al., 2015), but the specific context of selection appears to be critical. In the case of *PPM1D* mutations, *PPM1D* mutants are more “fit” only in the context of specific classes of chemotherapy, particularly those associated with DNA cross-links (e.g., cisplatin), DNA intercalation (e.g., doxorubicin), or topoisomerase-mediated DNA repair/replication (e.g., etoposide). *PPM1D* mutants have also been shown to demonstrate selective outgrowth in the context of cytarabine (Kahn et al., 2018). In contrast, microtubule inhibitors, such as vincristine, do not appear to confer a fitness advantage to *PPM1D* mutants. An alternative possibility is that specific agents directly induce mutagenesis, and cells that acquire the appropriate mutations in the *PPM1D* locus then persist. In this study, we did not have pre t-AML/t-MDS samples available and thus were unable to definitively address this distinction. Yet, the *PPM1D* VAF range of 0.02–0.47 in our patient cohort suggests that after acquisition of the *PPM1D* mutation in a single cell, there is likely some form of selection that results in expansion of the mutant into a detectable clone. Furthermore, other studies have found *PPM1D* mutations prior to, or in absence of, chemotherapy (Genovese et al., 2014, Gillis et al., 2017, Takahashi et al., 2017, Wong et al., 2018).

A key question that arises is whether *PPM1D* mutations contribute to leukemia development. *PPM1D* mutations are seen with much higher frequency in therapy-related AML and MDS than in *de novo* diseases (Figure 1). However, the VAF of mutant *PPM1D* in our t-AML/t-MDS cohort is low in the majority of cases, with a median of 0.05 and mean of 0.1, consistent with prior reports (Lindsley et al., 2017). This raises the question of whether *PPM1D* mutations can be leukemia-founding mutations or if they represent a co-existing sub-clone, or even a bystander clone, and whether *PPM1D* mutations mechanistically contribute to driving leukemia development. Future experiments will be necessary to address this thoroughly. Nevertheless, in a subset of cases in our cohort, *PPM1D* mutations consisted of a substantial portion of marrow cells (VAF >0.2); in six of those instances, we calculated the cancer cell fraction (CCF) of the mutation and observed that five out of six cases had a CCF of 1.0, indicating that many tumor cells carried the *PPM1D* mutation. Moreover, our PDX model with engrafted leukemic cells from a *PPM1D-*mutated t-AML sample provides further supportive evidence that *PPM1D* mutants can comprise a significant portion of diseased cells and may play a role in progression to leukemogenesis at least in some cases.

The fact that *PPM1D* mutations cause a gain-of-function and thus constitutively inhibit DNA damage activation of p53 is interesting given the role of p53 as a tumor suppressor. It is not clear from our data whether PPM1D is acting primarily through suppression of p53; however, PPM1D is also known to suppress numerous other components of the DNA damage response (reviewed Lu et al., 2008). The fact that *PPM1D* mutants do not completely phenocopy *TP53* mutations—for example, *PPM1D*-mutated t-AML/t-MDS patients do not appear to have the number of chromosomal abnormalities that *TP53* mutant t-MDS patients do (Lindsley et al., 2017)—suggests the mechanism may be more complex. We also note that *PPM1D* mutations have been observed in individuals with CH that are not reported to have been exposed to chemotherapy (Genovese et al., 2014). As more data on CH in the general population is accumulated, it will be important to determine whether *PPM1D* mutant clones can also rise via neutral drift or whether there are as-yet-unidentified environmental exposures that promote their relative survival. Nevertheless, it is important to note that *TP53* mutations provide stem cells an advantage in other contexts. Hematopoietic stem cells from mice with reduced levels of p53 were able to outcompete WT cells, particularly with age (Bondar and Medzhitov, 2010, Dumble et al., 2007).

Another study looking at cooperation among cells during embryonic development found that embryonic stem cells with *p53* or *Top1* knockdown completely supersede their WT neighbors without, remarkably enough, disrupting embryonic development (Dejosez et al., 2013). In that study, too, the success of p53-deficient cells was dependent on context: homogeneous populations of these cells show normal growth, and only in mixed populations do they expand disproportionately. Dejosez et al. (2013) proposed that these genes allow cells to cooperate with other cells in response to changes in the microenvironment. Once again, when considering evolutionary fitness, context is everything. It will be important to understand the very different environmental factors (fitness landscapes) that promote expansion of cells with distinct CH-associated mutations. The recent report of pre-leukemic expansion of Tet2-deficient cells driven by inflammatory cytokines after bacterial infection (Meisel et al., 2018) indicates there may be myriad relevant but as yet unidentified stressors.

In conclusion, *PPM1D* is clearly relevant to, although not sufficient for, the development of future hematological malignancies. Rising *PPM1D-*mutant clones were clearly associated with prior exposure to platinum agents and the topoisomerase inhibitor etoposide. This suggests that increased expression of PPM1D, either through the stabilizing truncating mutations or copy number gain, confers a selective advantage in the context of these cytotoxic therapies by causing the mutant cells to resist apoptosis. In the broader context of CH, we posit that mutations that differentially improve HSC survival, even without a particular impact on self-renewal, differentiation or proliferation, will increase the likelihood of that cell appearing as a CH clone, and also in its chance to accumulate further mutations that could be oncogenic. These studies underscore the importance of understanding specific treatment-mutation interactions in order to inform the choice of intervention for cancer patients and deriving prognostic implications when CH is detected.

## STAR★Methods

### Key Resources Table

**Table:** 

REAGENT or RESOURCE	SOURCE	IDENTIFIER
**Antibodies**		

Mouse monoclonal anti-WIP1 (F-10)	Santa Cruz	sc-376257; RRID:AB_10986000
Rabbit monoclonal anti-WIP1 (D4F7)	Cell Signaling	11901S
Mouse monoclonal anti-GAPDH (Clone 6C5)	Millipore	MAB374; RRID:AB_2107445
Rabbit polyclonal anti-P-p53 (S15)	Cell Signaling	9284S; RRID:AB_331464
Rabbit polyclonal anti-p53	Cell Signaling	9282S; RRID:AB_10693944
Rabbit polyclonal anti-P-Histone H2A.X (S139)	Cell Signaling	2577S; RRID:AB_2118010
Rabbit polyclonal anti-H2A.X	Cell Signaling	2595S; RRID:AB_10694556
eFluor 450 anti-mouse B220 (Clone RA3-6B2)	eBioscience	Cat# 48-0452-82; RRID:AB_1548761
eFluor 450 anti-mouse CD19 (Clone eBio1D3)	eBioscience	Cat# 48-0193-82; RRID:AB_2734905
eFluor 450 anti-mouse CD4 (Clone RM4-5)	eBioscience	Cat# 48-0042-82; RRID:AB_1272194
Pacific Blue anti-mouse CD8 (Clone 53-6.7)	BD Biosciences	Cat# 558106; RRID:AB_397029
eFluor 450 anti-mouse CD3 (Clone 17A2)	eBioscience	Cat# 48-0032-82; RRID:AB_1272193
eFluor 450 anti-mouse CD11b (Clone M170)	eBioscience	Cat# 48-0112-82; RRID:AB_1582236
eFluor 450 anti-mouse Gr1 (Clone RB6-8C5)	eBioscience	Cat# 48-5931-82; RRID:AB_1548788
eFluor 450 anti-mouse Ter119 (Clone TER-119)	eBioscience	Cat# 48-5921-82; RRID:AB_1518808
PE-Cy7 anti-mouse Sca-1 (Clone D7)	eBioscience	Cat# 25-5981-82; RRID:AB_469669
APC Cy7 anti-mouse cKit (Clone 2B8)	eBioscience	Cat# 47-1171-82; RRID:AB_1272177
Buv395 anti-mouse CD45.1 (Clone A20)	BD Biosciences	Cat# 565212; RRID:AB_2722493
APC anti-mouse CD45.2 (Clone 104)	eBioscience	Cat# 17-0454-82; RRID:AB_469400
PE anti-mouse CD150 (Clone TC15-12F12.2)	BioLegend	Cat# 115904; RRID:AB_313683
FITC anti-mouse CD48 (Clone HM48-1)	BD Biosciences	Cat# 11-0481-82; RRID:AB_465077
PE anti-mouse FcR II/III (Clone 2.4G2)	BD Biosciences	Cat# 553145; RRID:AB_394660
FITC anti-mouse CD34 (Clone RAM34)	BD Biosciences	Cat# 11-0341-82; RRID:AB_465021
PeCy7 anti-mouse B220 (Clone RA3-6B2)	BD Biosciences	Cat# 25-0452-82; RRID:AB_469627
PeCy7 anti-mouse CD11b (Clone M1/70)	BD Biosciences	Cat# 25-0112-82; RRID:AB_469588
PeCy7 anti-mouse Gr-1 (Clone RB6-8C5)	BD Biosciences	Cat# 25-5931-82; RRID:AB_469663
PE anti-human CD45 antibody	BioLegend	Cat# #304008; RRID:AB_314396
APC-conjugated anti-mouse CD45 antibody	BD Biosciences	Cat# 559864; RRID:AB_398672
Mouse FITC anti-BrdU (Clone B44)	BD Biosciences	Cat# 347583; RRID:AB_400327
APC Annexin V	BD Biosciences	Cat# 550475

**Biological Samples**		

Human primary t-AML sample (for patient-derived xenograft)	MD Anderson Cancer Center	N/A

**Chemicals, Peptides, and Recombinant Proteins**		

Cisplatin	Sigma Aldrich	Cat# C2210000
GSK2730371	Sigma Aldrich	Cat# SML1048
Etoposide	Sigma Aldrich	Cat# E1383
Doxorubicin	Sigma Aldrich	Cat# D1515
Annexin V Binding Buffer, 10X concentrate	BD Biosciences	Cat# 556454
7-AAD	BioLegend	Cat# 420403
Halt Protease and Phosphatase Inhibitor Cocktail (100X)	Thermo Scientific	Cat# 78440
Dimethyl sulfoxide (DMSO)	Sigma-Aldrich	Cat# 472301
Lipofectamine 2000	ThermoFisher	Cat# 11668027
Cell Counting Kit-8 (CCK-8)	Dojindo	Cat# CK04-11
Biomol Green	ENZO Lifesciences	Cat# BML-AK111-0250
Anti-Flag M2 affinity gel	Sigma Aldrich	Cat# F2426
P53 serine 15 phosphopeptide Ac-VEPPL(pS)QETFS-Amide)	China Peptides	N/A
3X Flag peptide	Sigma Aldrich	Cat# F3290
SpCas9 protein 3NLS	IDT	Cat# 1074181

**Critical Commercial Assays**		

MinElute PCR Purification Kit	QIAGEN	Cat# 28004
HiScribe T7 High Yield RNA Synthesis Kit	NEB	Cat# E2050S
RNA Clean & Concentrator-25	Zymo Research	Cat# D4013
KAPA HiFi HotStart ReadyMix (2X)	KAPA Biosystem	Cat# KK2601
Site-directed Mutagenesis Kit	Agilent Technologies	Cat#200523

**Experimental Models: Cell Lines**		

HEK293	ATCC	Cat# CRL-1573
MOLM13	DSMZ	Cat# ACC-554
OCI-AML3	DSMZ	Cat# ACC-582
OCI-AML2	DSMZ	Cat# ACC-99

**Experimental Models: Organisms/Strains**		

Mouse: *Ppm1d*^*R451X/+*^	This paper	N/A
Mouse: CD45.1 (B6.SJL-Ptprc^a^ Pepc^b^/BoyJ)	Jackson Laboratory	Stock #002014
Mouse: NSG (NOD.Cg-*Prkdc*^*scid*^*IL2rg*^*tm1Wjl*^/SzJ)	Jackson Laboratory	Stock# 005557

**Oligonucleotides**		

Custom primers and sgRNAs – Table S4	This paper	N/A

**Recombinant DNA**		

PPM1D-Flag-CMV-Neo-Bam	Fiscella et al., 1997	N/A
PPM1D D314A-Flag-CMV-Neo-Bam	This paper	N/A
PPM1D C478X-Flag-CMV-Neo-Bam	This paper	N/A
PPM1D E525X-Flag-CMV-Neo-Bam	This paper	N/A
PPM1D R458X-Flag-CMV-Neo-Bam	This paper	N/A
PPM1D R552X-Flag-CMV-Neo-Bam	This paper	N/A

**Software and Algorithms**		

FACSdiva v8.0.1	BD Biosciences	N/A
FlowJo X	FlowJo, LLC	https://www.flowjo.com/solutions/flowjo/downloads
Graphpad Prism 6.0	Graphpad Software	https://www.graphpad.com/
Adobe Illustrator CS6	Adobe	N/A
Snapgene 2.3.2	Snapgene	http://www.snapgene.com/
GenomeStudio 2.0	Illumina	https://support.illumina.com/downloads/genomestudio-2-0.html
Conumee R package 1.9.0	Bioconductor	http://bioconductor.org/packages/release/bioc/html/conumee.html
SPSS Software	IBM	https://www.ibm.com/analytics/spss-statistics-software

**Other**		

NEON Transfection System	ThermoFisher	N/A

### Contact for Reagent and Resource Sharing

Further information and requests for resources and reagents should be directed to and will be fulfilled by the Lead Contact, Margaret A. Goodell (goodell@bcm.edu).

### Experimental Model and Subject Details

#### Human subjects

The study population included patients diagnosed with therapy-related AML and therapy-related MDS, who were treated at MD Anderson Leukemia Clinic between 2010-2015. The patients were identified through the institutional medical records database. Inclusion criteria included prior diagnosis and treatment for at least one primary malignancy, with subsequent development of a therapy related myeloid neoplasm. Patients were excluded from analysis if they had a primary malignancy that was not treated with chemotherapy, radiation therapy, or systemic agents. In total, 156 patients met criteria for the t-AML/t-MDS cohort. See Tables S1 and S2 for patient characteristics (in brief, the cohort included 84 males and 72 females, with a median age of 67 (range 58-72)). All bone marrow and peripheral blood samples were obtained at the time of or shortly after t-AML or t-MDS diagnosis, with written consent from patients and approval for research use by the MDACC Institutional Review Board. The control cohort (for comparison of genomic landscape) consists of 228 patients diagnosed with *de novo* AML or MDS, with no prior exposure to chemotherapy and radiation therapy, at MD Anderson within the same time period. Targeted-capture sequencing and PPM1D re-sequencing by the same protocol described below had been previously performed on these *de novo* cases, and the results were utilized for comparison with our t-MN cohort. Clinical characteristics such as blood counts were obtained at the time of t-AML/t-MDS diagnosis. Clinical data, including prior primary malignancy, exposure to cytotoxic and radiation therapy, treatment protocols received for t-MN, response rates and overall survival were curated from clinical chart review of each patient’s medical history. Smoking history was self-reported. In some categories, the total number reported in tables or graphs may be less than 156, if there was insufficient or missing data. Lastly, it was noted that the association between sex and *PPM1D* mutational status was not statistically significant (*PPM1D* mutated cases included 17 males and 14 females, p = 0.90). Thus, we did not pursue further analyses on the influence of sex on findings from this patient cohort.

#### Cell Lines

MOLM13 cells were cultured in RPMI (GIBCO), OCI-AML2 and OCI-AML3 cells in MEM alpha (GIBCO), and HEK293 cells in DMEM (GIBCO), all supplemented with 10% FBS (Corning) and 1% Pen/Strep (GIBCO). Cells were cultured in a humidified atmosphere containing 5% CO_2_ at 37°C. All cell lines tested negative for mycoplasma using a PCR-based method. The HEK293 line was purchased directly from ATCC, where authentication has been performed. The OCI-AML2, OCI-AML3, and MOLM13 lines were not authenticated; however, all experiments involve comparisons between isogenic control and mutant lines derived from the same parent line. The sex of the cell lines is as follows: HEK293 – female, MOLM13 – male, OCI-AML2 – male, OCI-AML3 – male.

#### Mice

All mice were housed in AAALAC-accredited, specific-pathogen-free animal care facilities at Baylor College of Medicine, and all procedures were approved by the BCM Institutional Animal Care and Use Committee. Mice of both sexes were used, and experimental mice were separated by sex and housed with 5 mice per cage. All mice were immune-competent and healthy prior to the experiments described. Tail snips were performed to obtain tissue for genotyping at the time of weaning. Otherwise, no experimental procedures, tests, or drugs were administered prior to the studies described.

##### Generation of the Ppm1d R451X mouse model

The *Ppm1d1* R451X knock-in mouse model was generated with CRISPR-Cas9 in collaboration with the Mouse Embryonic Stem Cell Core and Genetically Engineered Mouse Core at Baylor College of Medicine. A sgRNA (sequence GTCCCAGCTGAGATAGCTAG) was used to induce a double stranded break in the endogenous allele close to amino acid 451. The single-stranded oligo template for homology-directed repair was designed to introduce a premature stop codon after amino acid 450 (via a point mutation), with a GGGGS linker and 3x FLAG tag preceding the stop codon. This construct was flanked by proximal and distal homology arms. The full sequence of the synthesized ssoligo was: AGAAGTTTTTAGAGGTCCCAGCTGAGATAGCTGGTGGTGGCGGTTCAGACTACAAAGACCATGACGGTGATTATAAAGATCATGACATCGACTACAAGGATGACGATGACAAGTGAGGGAATATCCAGACTGTAGTGATGACCTCAAAAGACTCAGAGACACTTGAAGAAAATTGCCCCAAAGCCCTGACTTTAAGG.

A total of 200 C57BL/6 embryos were cytoplasmically injected with the following in 60uL of 1X sterile PBS: Cas9 (wild-type), 100ng/ul; 1 sgRNA, 20ng/ul; 1 ssOligo, 100 ng/ul. The injected embryos were transferred into the oviducts of ICR recipient mice. Twenty-three founder pups were born, and 3 correctly incorporated the 3xFLAG tag and stop codon, as determined by PCR genotyping and verified by Sanger Sequencing. The sequence was visualized with the sequence alignment function in the Snapgene software. These mosaic R451X founder mice were crossed with wild-type C57BL/6 to obtain heterozygous F1 mice. This *Ppm1d* ^R451X/+^ line was subsequently maintained by further backcrosses with wild-type C57BL/6 mice. Hematopoiesis in both male and female *PPM1D*^R451X/+^ 8-week old mice was characterized, and no significant differences were observed in peripheral blood composition, or proportions of progenitors and stem cells between the two sexes.

##### Mouse genotyping

Mouse DNA was extracted from mouse tail by using DirectPCR Lysis Reagent (Viagen). Extracted DNA was used for PCR genotyping. The specific PCR primers for the *Ppm1d* R451X mouse were: AGGCTGAGCTCTAAGGACCA (forward) and ATTGGCTGGAGGGGTTCTTT (reverse). The PCR product was 396 bp for the flag-tagged *Ppm1d* truncated allele, and 315 bp for the wild-type allele.

#### Patient Derived Xenograft model

The t-AML PDX model was generated using NOD.Cg-*Prkdc*^*scid*^ *IL2rg*^*tm1Wjl*^/SzJ (NSG) mice, in accordance with The University of Texas MD Anderson Cancer Center IACUC guidelines (protocol #00000884-RN02) and NIH guidelines.

Frozen primary t-AML cells were thawed, and cells were intravenously transplanted into sub-lethally irradiated NSG mice (250cGy). 5x10^5^ total cells were transplanted into each mouse, and peripheral blood analysis was performed every two weeks following transplantation, to monitor the disease burden. Briefly, peripheral blood samples were treated with red cell lysis buffer, and the cells were washed prior to staining with PE-conjugated anti-human CD45 antibody (BioLegend #304008) or APC-conjugated anti-mouse CD45 antibody (BD Bioscience #559864), and DAPI (ThermoScientific #62248). Flow-cytometry was performed to assess the relative percentages of murine and human CD45^+^ cells and to track human CD45 (hCD45) engraftment in the peripheral blood over time. Animals were sacrificed and bone marrow was harvested when the percentage of hCD45^+^ cells reached 20%–30% of the peripheral blood. Serial transplantation was performed with the harvested bone marrow, which was determined to have greater than 90% hCD45^+^ cells. 1x10^6^ bone marrow cells were serially transplanted into sub-lethally irradiated recipient NSG mice (250cGy). An aliquot of bone marrow cells was analyzed by next-generation sequencing to determine *PPM1D* variant allele frequency (VAF). The secondary recipient mice were monitored for clinical signs of illness, and peripheral blood was routinely analyzed for expansion of leukemic hCD45-expressing cells. When the percentage of hCD45^+^ cells exceeded 30% of peripheral blood cells, the mice were sacrificed. Bone marrow and splenic cells from these secondary recipient mice were harvested, and analyzed by next-generation sequencing to determine *PPM1D* VAF.

### Method Details

#### DNA sequencing

Genomic DNA was extracted from the diagnostic t-MN bone marrow samples using the Autopure extractor (QIAGEN/Gentra, Valencia, CA), according to the manufacturer’s instructions. A customized 295-gene panel was utilized for targeted sequencing, as previously described (Morita et al., 2018). As *PPM1D* was not included on the first gene panel, we re-visited all 156 t-MN samples and performed targeted PCR amplification of exon 6 of *PPM1D*, utilizing 2 sets of PCR primers designed to yield overlapping amplicons that cover the entirety of the exon. The 2 sets of PCR primers used were (5′ to 3′): Set 1 Fwd = TGCATAGATTTGTTGAGTTCTGG, Set1 Rev = AGGCCAATTGGAAGGCTATT, and Set 2 Fwd = ATTGCGCTAAAGCCCTGAC and Set 2 Rev = TCTTCTGGCCCCTAAGTCTG). The PCR amplicons were purified with QIAGEN MinElute columns, and 300ng was submitted for library preparation and next generation sequencing on the Illumina Hiseq2000 platform. As previously described (Takahashi et al., 2017), raw sequencing data from the Illumina platform were converted to a fastq format and aligned to the reference genome (hg19) using the Burroughs-Wheeler Aligner (BWA). The aligned BAM files were subjected to mark duplication, re-alignment, and re-calibration using Picard and GATK. Preprocessed BAM files were then analyzed to detect single nucleotide variants (SNV) and small insertions and deletions (indels) using MuTect and Pindel algorithms, respectively, against virtual normal sequence developed in-house. Variants with VAF equal to or greater than 0.02 were retained.

#### SNP array for PPM1D copy number alteration

We used Illumina Infinium Omni 5-4 (N = 91) and Omni 2.5-8 (N = 29) bead chips to assess DNA copy number profiles of 120 t-AML/t-MDS marrow samples. Genomic DNA was processed by whole genome amplification, enzymatic fragmentation and sequence-specific hybridization to the bead chips at 48 degree for 16-24 hr. After target hybridization to the bead arrays, primers hybridized to the template were extended by enzymatic allele-specific primers, stained with two-color single base extension biochemistry and the chips were scanned on the iScan (Illumina Inc.) IDAT files generated from the scanner were uploaded onto GenomeStudio 2.0 software. Copy number profiles were generated using Conumee R package in Bioconductor (http://bioconductor.org/packages/release/bioc/html/conumee.html). Raw intensity values from two alleles (A and B allele) were combined and used as a raw input. Subsequent fitting, binning and segmentation were performed with the program’s default setting.

#### Generation of PPM1D mutant cell lines

We generated the *PPM1D* mutant cell lines in MOLM13, HEK293, OCI-AML2, and OCI-AML3 using the RNP-based CRISPR/Cas9 delivery method, and sgRNAs were synthesized as previously described (Brunetti et al., 2018, Gundry et al., 2016). In brief, the CRISPRscan algorithm was used to identify proto-spacer sequences in exon 6 of *PPM1D*. We selected a pair of sgRNAs that creates an out-of-frame deletion in exon 6 (guide sequences from 5′ to 3′ are GGGTCCTTAGAATTCACCCT and GGAAGGCATTGCTACGAACC). For synthesis of sgRNAs, full-length DNA templates were produced by overlap PCR, and the PCR products were purified with the MinElute PCR purification kit (QIAGEN), followed by *in vitro* transcription with the HiScribe T7 High Yield RNA Synthesis Kit (NEB) per manufacturer’s protocol. The *in vitro* transcription products were purified using the RNA Clean & Concentrator-25 and eluted in nuclease-free water, following the manufacturer’s instructions. Cas9-sgRNA RNPs were then generated by incubating 1ug of Cas9 protein (IDT) with 1ug of the sgRNA pair for 15 minutes at room temperature, prior to electroporation with 2 x10^5^ cells in Buffer R using the Neon Transfection System (ThermoFisher). Electroporation conditions used were 1500V, 30ms, 1 pulse for HEK293 and 1350V, 35ms, 1 pulse for MOLM13, OCI-AMl2, and OCI-AML3. Single cell clones were isolated and screened for the deletion created by the sgRNA pair by PCR (forward primer = TGCATAGATTTGTTGAGTTCTGG, reverse primer = TGGTTCTGGATCTTTTGAGGGT). Positive clones were Sanger-sequenced for verification of an exon 6 truncation. Sequencing results revealed that the HEK293 mutant clone had a truncation at amino acid 431 and the MOLM13 mutant clone had a truncation at amino acid 440. For the isogenic control cell line for MOLM13, we electroporated cells with a pair of in-frame sgRNAs targeting the last exon of *ENAM*, a gene that encodes enamel in the teeth (sgRNA sequences are GGATGATGTGTCCACGCTG and GGGAACTGGCTTCAGGAAA).

#### Immunoblotting

Cells or minced mouse tissue were lysed with 1x RIPA buffer supplemented with the Halt Protease and Phosphatase inhibitor cocktail (ThermoFisher) for 30 minutes at 4°C. Protein concentration was quantified using the Pierce BCA protein assay kit (Thermo Fisher), and lysates were then boiled at 95C in 1x Laemmli (Bio-Rad) for 5 mins. The proteins were separated by SDS-PAGE on 4%–15% gradient gels (Bio-Rad), and transferred onto nitrocellulose membranes (Bio-Rad). For the blot in Figure 3A, duplicate sets of samples were run for parallel blotting. After 1 hour of blocking, membranes were incubated overnight at 4°C with the following primary antibodies: mouse anti-PPM1D (F-10, Santa Cruz, 1:1000, for detection of human PPM1D), rabbit anti-p53 ser15 (#9284, Cell Signaling, 1:1000), rabbit anti-total p53 (#9282, Cell Signaling, 1:1000), rabbit anti-γH2AX ser139 (#2577, Cell Signaling, 1:1000), rabbit anti-total H2AX (#2595, Cell Signaling, 1:1000), mouse anti-GAPDH (MAB374, Millipore, 1:2000), rabbit anti-PPM1D (D4F7, Cell Signaling, 1:1000, for detection of mouse PPM1D). This was followed by secondary antibody incubation with anti-mouse or anti-rabbit horseradish peroxidase-conjugated secondary antibody (Santa Cruz), and imaging on the Bio-Rad ChemiDoc platform.

#### *In vitro* phosphatase assay

The wild-type PPM1D-Flag vector (PPM1D-Flag-CMV-Neo-Bam) was a gift from Dr. Ettore Appella. Phosphatase dead D314A (PD), C478X, E525X, R458X, and R552X mutants were created using a site directed mutagenesis kit (Agilent Technologies) and the wild-type PPM1D-Flag construct as the backbone. The individual constructs were transfected into 293 cells using Lipofectamine 2000 (Thermo Fisher) and PPM1D proteins were immunoprecipitated 48 hours later using anti-Flag M2 affinity gel (Sigma Aldrich). Immunoprecipitates were washed once in TBS and 3 times in PP2C buffer (50mM Trish-HCl pH 7.5, 0.1 mM EGTA, and 0.02% β-mercaptoethanol). Proteins were released from the beads using 3x Flag peptide (Sigma Aldrich) diluted in PP2C buffer. Equal volumes of immunoprecipitated PPM1D were incubated with 30mM MgCl_2_ and 100uM p53 serine15 phosphopeptide (Ac-VEPPL(pS)QETFS-Amide). Reactions were incubated at 25°C for 1 hour. Free phosphate was measured with the addition of Biomol Green (ENZO Lifesciences). Absorbance was read at 630nm in a Victor 2 multilabel 96-well plate reader (Perkin Elmer). The results were normalized to enzyme input based on densitometric analysis of western results for the immunoprecipitated proteins.

#### Chemotherapy dose-response experiments

The stock solutions of cisplatin (Sigma, C2210000) and GSK2730371 (Sigma, SML1048) were prepared in water, and etoposide (Sigma, E1383) and doxorubicin (Sigma, D1515) were prepared in DMSO. Isogenic *PPM1D* WT and mutant clones generated from MOLM13, OCI-AML2, and OCI-AML3 were seeded at 5000 cells per well in 96-well flat-bottom plates. An 8-point, 3-fold serial dilution of the drugs was added to the plates, to a final volume of 100uL per well (n = 3 replicates for each concentration). Cell viability was measured after 48 hours by the addition of Cell Counting Kit-8 (WST-8) reagent (Dojindo), 10uL per well, followed by incubation at 37°C for 4 h. Absorbance values of the wells were recorded with microplate reader (Perkin Elmer) at 450 nm. The viability readings were normalized to that of cells treated with vehicle alone. The resulting data were analyzed by using the dose-response function in Prism 6 (Graphpad Software, San Diego, CA). Dose-response curves were created and the concentration corresponding to the IC_50_ was determined.

#### Detection of apoptosis

Annexin V-APC and 7-AAD staining was used for the quantitation of early and late apoptotic cells. MOLM13 cells were treated with 1uM cisplatin (+/− 24nM GSK2830371) for 72 hours, washed and resuspended in 100uL of 1x annexin binding buffer (BD Biosciences) with annexin V-APC (BioLegend, 3:100 concentration) and 7-AAD (BioLegend, 3:100 concentration) for 20 minutes. Cells were analyzed by flow cytometry (LSRII, Becton Dickinson) with the FACSdiva software (BD Biosciences). A minimum of 10,000 cells was analyzed per sample, and data was visualized using the FlowJo software.

#### Cell cycle analysis

*PPM1D* wild-type and mutant cells were treated with 750nM cisplatin (or vehicle) for 24 hours. Prior to harvest, the cells were pulsed with BrdU for 1 hr at a 10uM concentration in the media. The cells were then washed, pelleted, and fixed with 70% ethanol in HBSS overnight for BrdU cell cycle analysis. The next day, fixed cells were spun down and the fixative liquid was aspirated. Cells were resuspended in denaturation solution (2N HCl with 0.5% Triton X in PBS) for 30 minutes at room temperature, then pelleted and resuspended in neutralization solution (0.1M Na_2_B_4_O_7_ in water, pH = 8.5). The cells were pelleted again, and incubated with FITC Anti-BrdU antibody (10uL in 50uL of 1% BSA, 0.5% Triton X antibody solution) for 1 hr at room temperature. Cells were then washed with 1% BSA in PBS, spun down, and resuspended in 50ug/mL propidium iodide (+10ug/mL RNase) in PBS. Cells were then analyzed by flow cytometry (LSRII, Becton Dickinson). 3 independent experiments were performed, in triplicate.

#### *In vitro* cell competition

Isogenic MOLM13 and GFP-expressing MOLM13 cells were a gift from Dr. Karen Rabin. *PPM1D* mutant MOLM13 cells (clonal line generated as described earlier) were mixed with control (*ENAM*-edited) GFP-expressing MOLM13 cells. Specifically, 200k *PPM1D* mutant cells were mixed with 800k control cells, for a total of 1 million cells per 6-well (in triplicates). Cells were either resuspended in 2mL of media with 1uM cisplatin, 1uM cisplatin with 18nM of GSK2830371, or vehicle (water). Every 4 days, cells were split 1:2, and the resuspended in media with fresh drug (or vehicle). Split cells were analyzed by flow cytometry to track the percentage of *PPM1D* mutant GFP-negative cells. Three independent experiments were performed, with technical triplicates. In the reciprocal experiment, clonal lines of CRISPR-generated *PPM1D-*mutant GFP-expressing MOLM13 cells were mixed with control GFP-negative MOLM13 cells in a 10:90 ratio and treated as above. The dosage used for the other chemotherapy agents (also refreshed every 4 days as above) are as follows: doxorubicin - 20nM, etoposide - 250nM, and vincristine - 1nM.

#### Murine bone marrow transplantation

The *Ppm1d* R451X mice (bearing the CD45.2 surface alloantigen) were crossed to wild-type mice (bearing the CD45.1 surface alloantigen) (B6.SJL-*Ptprc*^*a*^ *Pepc*^*b*^/BoyJ) for one generation to yield offspring (expressing both CD45.1 and CD45.2 alloantigens) that were either *Ppm1d* R451X heterozygotes or wild-type. For the competitive bone marrow transplantations, donor bone marrow from age and sex-matched 6 to 8-week old R451X or wild-type control littermates (CD45.1/CD45.2) were mixed with bone marrow from wild-type mice (CD45.1) in a 20:80 ratio, with a total of 3x10^6^ cells transplanted into each recipient mouse. Specifically, 6x10^5^ *Ppm1d* R451X or control cells (CD45.1/CD45.2) were mixed with 2.4x10^6^ wild-type cells (CD45.1), and retro-orbitally injected into 6- to 10-week old lethally irradiated CD45.1 recipient mice (split-dose of 1100cGy total, separated by 4 hours). Secondary transplantation was performed by transplanting 3 × 10^6^ bone marrow cells from the recipients of the first competitive transplant into lethally irradiated (1100cGy) secondary recipients.

Peripheral blood was collected at 4 weeks following transplant to determine engraftment and to establish the baseline chimerism. In the whole bone marrow competitive transplants, recipient mice were randomly allocated into treatment groups, and treated with either cisplatin (4mg/kg, intraperitoneal) or vehicle (water) once a week starting at 4 weeks post-transplant, for 5 consecutive weeks. Chimerism in the peripheral blood was monitored weekly by flow cytometry (LSRII, Becton Dickinson) in the first 8 weeks, and bi-weekly thereafter. The following antibodies were used for peripheral blood staining: APC-conjugated CD45.2, FITC-conjugated CD45.1, PeCy7-conjugated Gr-1, CD11b, B220, and PB-conjugated CD4, CD8, B220 (eBioscience). At 13 weeks post-transplant, several recipients from the competitive transplant cohort were sacrificed for analysis of the bone marrow and for serial transplantation.

Briefly, whole bone marrow cells were obtained by crushing the long bones (tibias and femurs) with a mortar and pestle in Hank’s buffered salt solution (HBSS), supplemented with 10mM HEPES (GIBCO) and 2% heat-inactivated bovine serum (Corning). Cells were filtered through a 40 μm cell strainer (ThermoFisher Scientific) to obtain a single-cell suspension. For analysis of populations in the bone marrow, the following antibodies were used: CD4-eFluor450 (1:100), CD8-PB (1:100), CD3-eFluor450 (1:100), Ter-119-eFluor450 (1:100), CD11b-eFluor450 (1:100), Gr-1-eFluor450 (1:100), B220-eFluor450 (1:100), CD19-eFluor450 (1:100), Sca1-APC Cy7 (1:100), cKit-PeCy7 (1:100), FcR II/III-PE (1:100), CD34-FITC (1:100), CD48-FITC (1:200), CD150-PE (1:100), CD45.2-APC (1:100), and CD45.1-BUV395 (1:100). All monoclonal antibodies were from BD Biosciences or eBioscience.

The LSRII/Fortessa cell analyzer was used for data acquisition, and data analysis was performed using the FlowJo software.

The treatment regimens for the additional chemotherapy agents are as follows: doxorubicin (3 rounds of 2mg/kg IP x 3 days, with 21 days between each round) and vincristine (8 rounds of 0.1mg/kg IP, once weekly).

The mouse studies were performed without blinding of the investigator and no animals were excluded from the analysis, with the exception of recipient mice that did not successfully engraft following transplant, or mice that were not used due to human-errors during the experiment. These numbers were minimal. With mouse bone marrow transplant experiments, 5-10 recipient mice per experimental group is a well-accepted sample size that is used by numerous studies in the field and in this study. This cohort size is typically sufficient for statistical power.

### Quantification and Statistical Analysis

Statistical analyses of the clinical data were performed with the SPSS software. Associations between pairs of categorical variables were assessed using Fisher’s exact test or Pearson’s Chi square test. Survival following t-MN diagnosis was assessed by Kaplan-Meier survival analysis, and Mantel Cox log rank test was performed to determine statistical significance. P values were interpreted as statistically significant if less than 0.05, unless otherwise stated. See also Figure Legends and the Results section for more details.

In the rest of the manuscript, all data are expressed as the mean ± SD, unless otherwise stated. Statistics were calculated and figures were generated with Graphpad Prism 6. The statistical significance of the differences between two groups was calculated using unpaired Student’s t test (two-sided), without assuming equal standard deviations. Statistical details are described in the Figure Legends, including the number of replicates or animals per group (denoted by “n”), as well as p values where relevant. ^∗^p < 0.05, ^∗∗^p < 0.01, ^∗∗∗^p < 0.001, ^∗∗∗∗^p < 0.0001.

## Supplemental Information

Document S1. Figures S1–S7 and Tables S1–S4

Document S2. Article plus Supplemental Information
