# Supplementary material for: PPM1D Mutations Drive Clonal Hematopoiesis in Response to Cytotoxic Chemotherapy
Source: Cell Stem Cell. 2018 Nov 1;23(5):700–713.e6. doi: 10.1016/j.stem.2018.10.004 (PMC6224657; doi:10.1016/j.stem.2018.10.004)
Supplement: Document S1. Figures S1–S7 and Tables S1–S4 [file mmc1.pdf]

Cell Stem Cell, Volume *23*

Supplemental Information

PPM1D Mutations Drive Clonal Hematopoiesis in Response to Cytotoxic Chemotherapy

Joanne I. Hsu, Tajhal Dayaram, Ayala Tovy, Etienne De Braekeleer, Mira Jeong, Feng Wang, Jianhua Zhang, Timothy P. Heffernan, Sonal Gera, Jeffrey J. Kovacs, Joseph R. Marszalek, Christopher Bristow, Yuanqing Yan, Guillermo Garcia-Manero, Hagop Kantarjian, George Vassiliou, P. Andrew Futreal, Lawrence A. Donehower, Koichi Takahashi, and Margaret A. Goodell

A

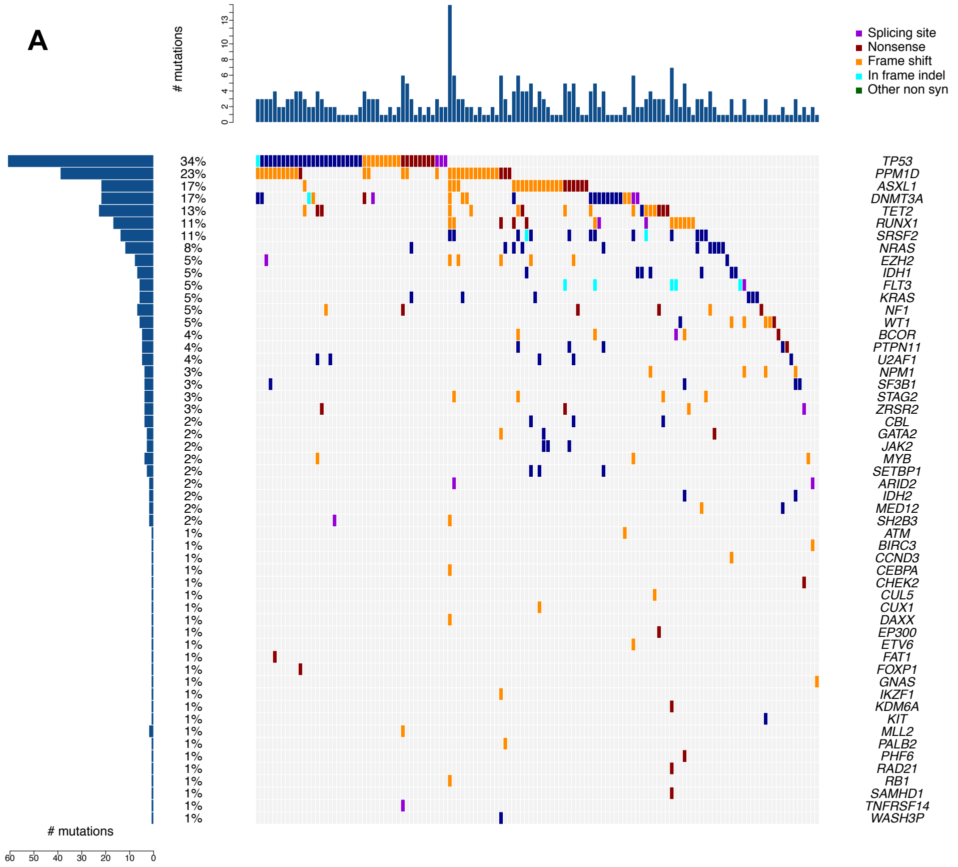

B

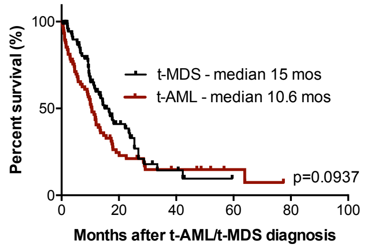

	n	Median, (95% Lower-Upper)
t-MDS	79	12.5 (10.8-19.2)
t-AML	77	10.6 (8.6-12.6)

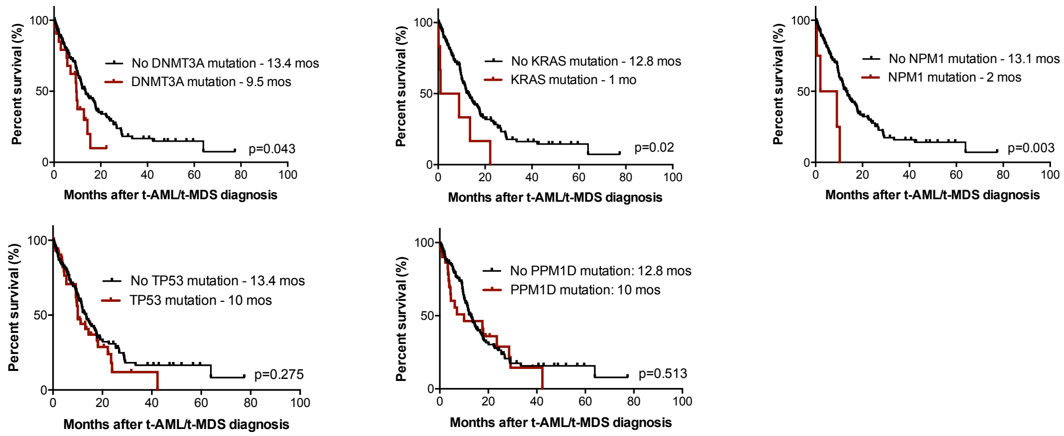

C

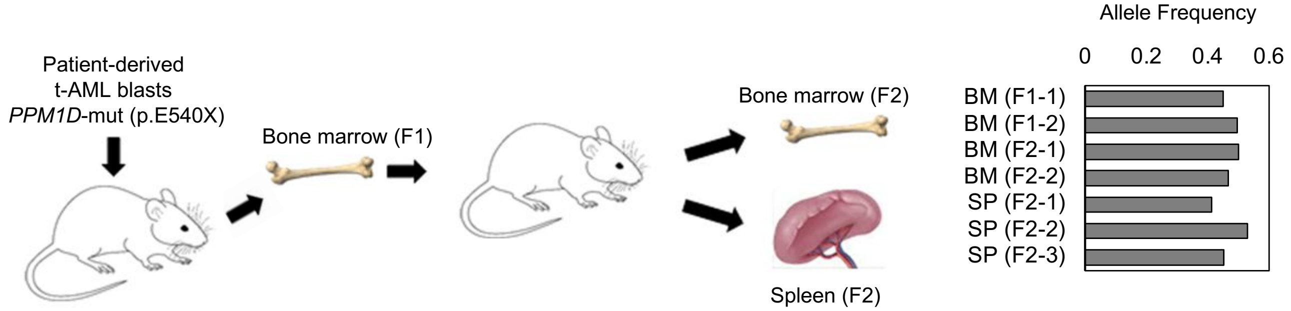

Figure S1. Additional mutation-associated data from the t-AML/t-MDS cohort (Related to Figure 1). (a) Mutational landscape of myeloid-neoplasm (MN) associated genes in t-AML/t-MDS and pairwise analysis of mutational associations. The number of mutations identified per individual is depicted by the histogram on the top. Mutation co-occurrence by individual is shown below, color-coded by mutation class, as indicated in the legend. **(b)** Kaplan Meier curves comparing overall survival between t-AML patients (n=77, depicted in red) and t-MDS patients (n=79, depicted in black) (hazard ratio, 1.39; 95% CI, 0.949-2.063; P=0.094). Tick marks indicate censored data. **(c)** Kaplan Meier curves for *TP53* and *PPM1D* as well as mutated genes with significantly worse overall survival (*DNMT3A*, *KRAS,* and *NPM1*, p=0.043, 0.02, and 0.003, respectively). **(d)** A patient-derived xenograft (PDX) model was generated by transplanting cells from a *PPM1D* mutated t-AML sample (E450X with VAF of 0.465) into two recipient immunodeficient NSG mice (denoted as F1). Following engraftment, bone marrow from each mouse was harvested for serial transplantation into secondary recipient mice (denoted as F2). Targeted next-generation sequencing was performed to determine *PPM1D* VAF in both the F1 bone marrow and the F2 spleen and bone marrow.

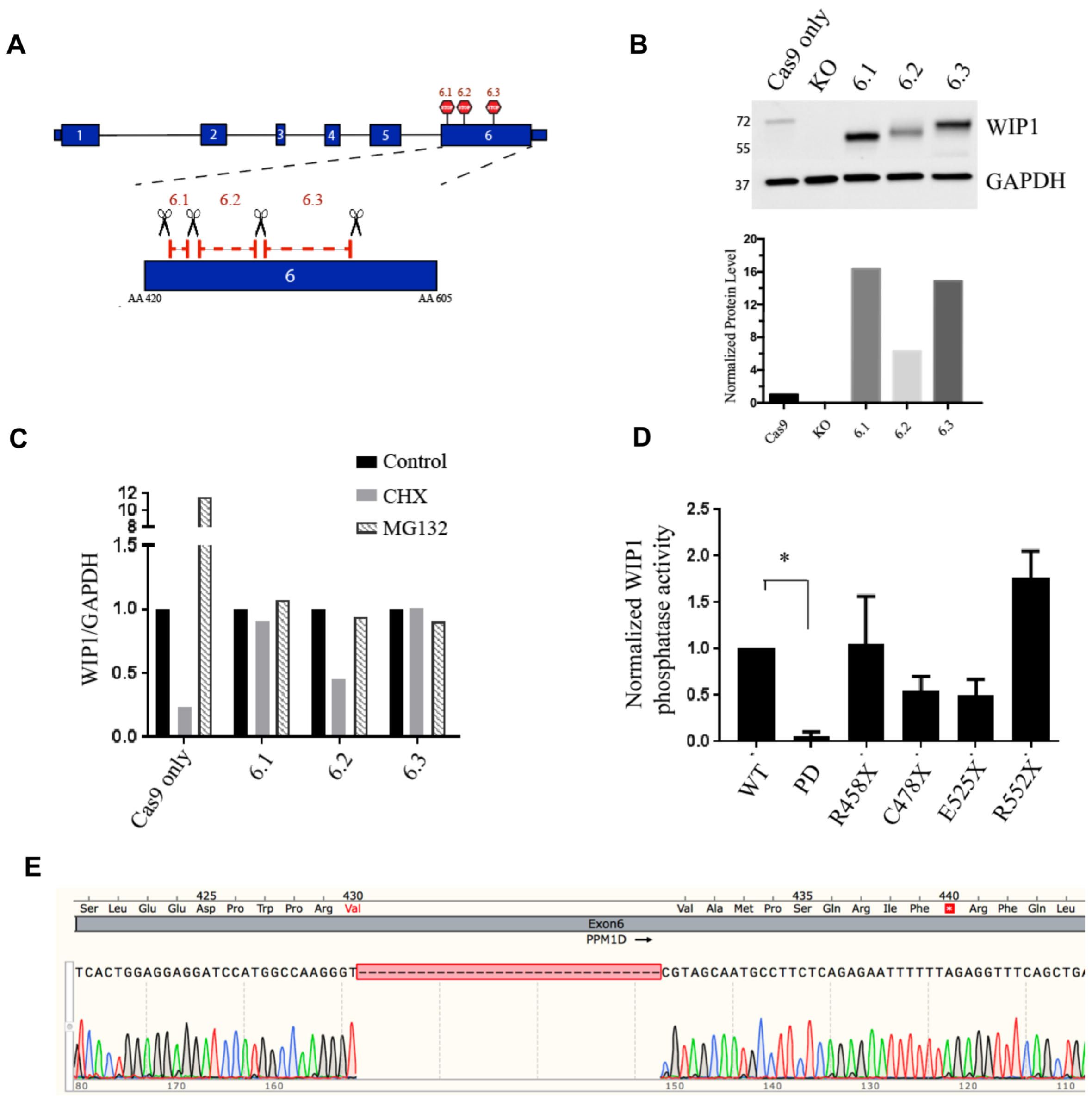

Figure S2. Generation of the *PPM1D* mutant cell lines and evaluation of protein stability and phosphatase activity (Related to Figure 3). (a) CRISPR-Cas9 editing strategy. Three different truncation mutants were generated in exon 6 of *PPM1D* (6.1, 6.2, and 6.3) using pairs of gRNAs to create out-of-frame deletions. Mutants were generated in HEK293, which is wildtype for *PPM1D* and *TP53*. The control line is Cas9 only without sgRNA. **(b)** Western blot of the bulk CRISPR-generated mutants demonstrate increased PPM1D protein levels at baseline in all three truncation mutants. Quantification of the bands reveals up to a 16-fold increase in mutant PPM1D protein level, compared to the Cas9 control. The KO line is a negative control (with a truncation in exon 1 of *PPM1D*). **(c)** Treatment of the PPM1D mutants with cyclohexamide and MG132 reveals truncated PPM1D has greater stability than the full-length PPM1D. **(d)** *In vitro* phosphatase assay performed with a synthetic p53 (serine 15) phosphopeptide reveals no significant difference in phosphatase activity between truncated and full-length PPM1D. **(e)** Sanger sequencing trace of exon 6 in the CRISPR-generated clonal *PPM1D*-truncated line (MOLM13). The out-of-frame deletion created by the pair of sgRNAs is depicted by the red dashed line.

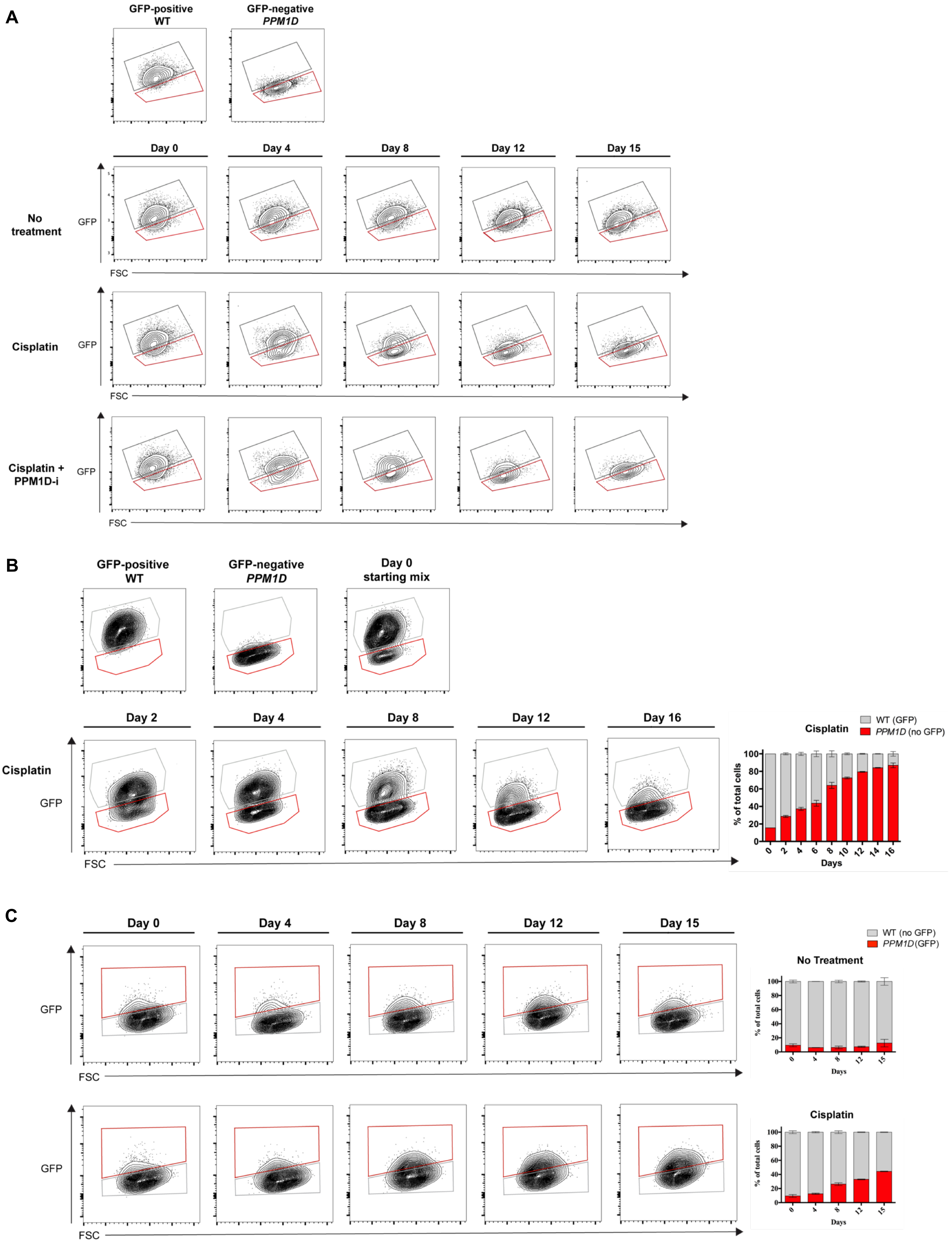
A
GFP-positive WT
GFP-negative PPM1D
Day 0
Day 4
Day 8
Day 12
Day 15
No treatment
Cisplatin
Cisplatin + PPM1D-i
GFP
FSC
B
GFP-positive WT
GFP-negative PPM1D
Day 0 starting mix
Day 2
Day 4
Day 8
Day 12
Day 16
Cisplatin
GFP
FSC
WT (GFP)
PPM1D (no GFP)
% of total cells
Days
C
Day 0
Day 4
Day 8
Day 12
Day 15
GFP
FSC
WT (no GFP)
PPM1D(GFP)
No Treatment
Cisplatin
% of total cells
Days

Figure S3. Flow cytometry plots showing the gating scheme for *in vitro* competition experiments (Related to Figure 3). (a) In the *in vitro* competition experiment, GFP-negative *PPM1D* mutant cells and GFP-positive *PPM1D* WT cells were competed at a starting 20:80 ratio, respectively. Representative flow plots (corresponding to Figure 3D) of the GFP-positive and GFP-negative populations are shown for the individual starting cell lines, as well as at time-points after the cell lines were mixed at a 20:80 ratio on Day 0. The GFP-positive WT population is indicated by the gray-colored gate, and the GFP-negative *PPM1D* mutant population is indicated by the red-colored gate. The mixed cells were subjected to three different conditions, as shown (NT = no treatment, Cis = 1uM cisplatin treatment, Cis + PPM1Di = 1uM cisplatin treatment with 18nM of GSK2830371). Each condition was performed in triplicate. **(b)** An independent experiment of the *in vitro* competition between GFP-positive *PPM1D* WT cells and GFP-negative *PPM1D* mutant was performed in triplicate. Flow cytometry analysis was performed every 2 days. Representative flow plots are shown for select time-points. A summary graph of the competition results is shown. **(c)** *In vitro* competition with reciprocal GFP labeling, where GFP-positive *PPM1D* mutant cells and GFP-negative *PPM1D* WT MOLM13 cells were mixed at a starting 10:90 ratio, respectively on Day 0. Representative flow plots at time-points are depicted, along with a summary graph.

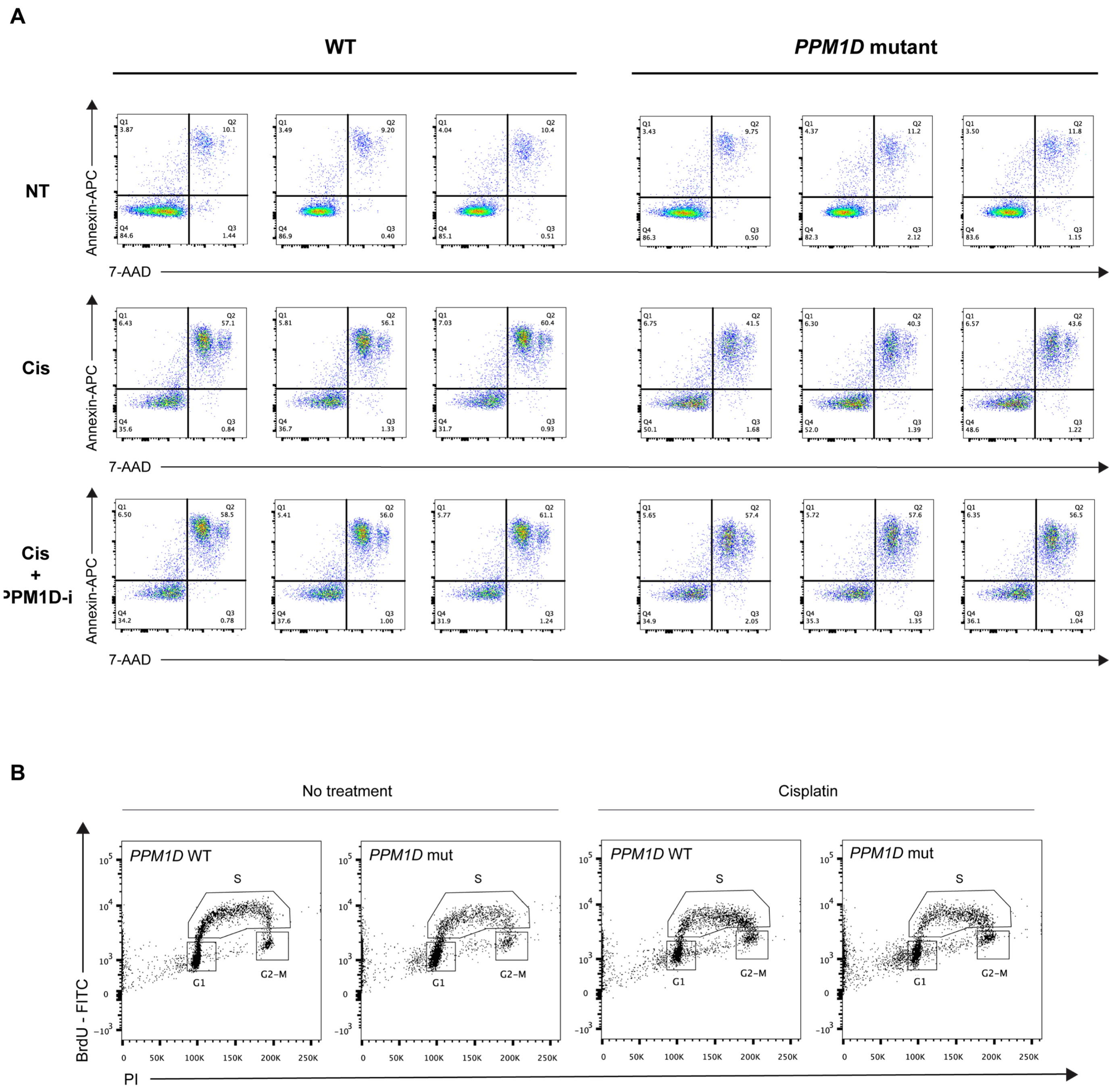

Figure S4. Flow cytometry plots showing the gating scheme for the apoptosis and cell cycle experiments (Related to Figure 3). (a) In the apoptosis assays, annexin V/7-AAD staining was performed in triplicate for each condition. 7-AAD is shown on the x-axis and Annexin V-APC on the y-axis of the flow cytometry plots. The data shown is representative of three independent experiments. **(b)** Representative flow cytometry plots for the BrdU cell cycle analysis are shown for WT and *PPM1D* mutant cells (+/- cisplatin treatment). Propidium iodide (PI) is shown on the x-axis and BrdU-FITC is shown on the y-axis. The gates denote the G1, S2, and G2/M populations. The experiment was performed in four independent experiments, each in triplicate.

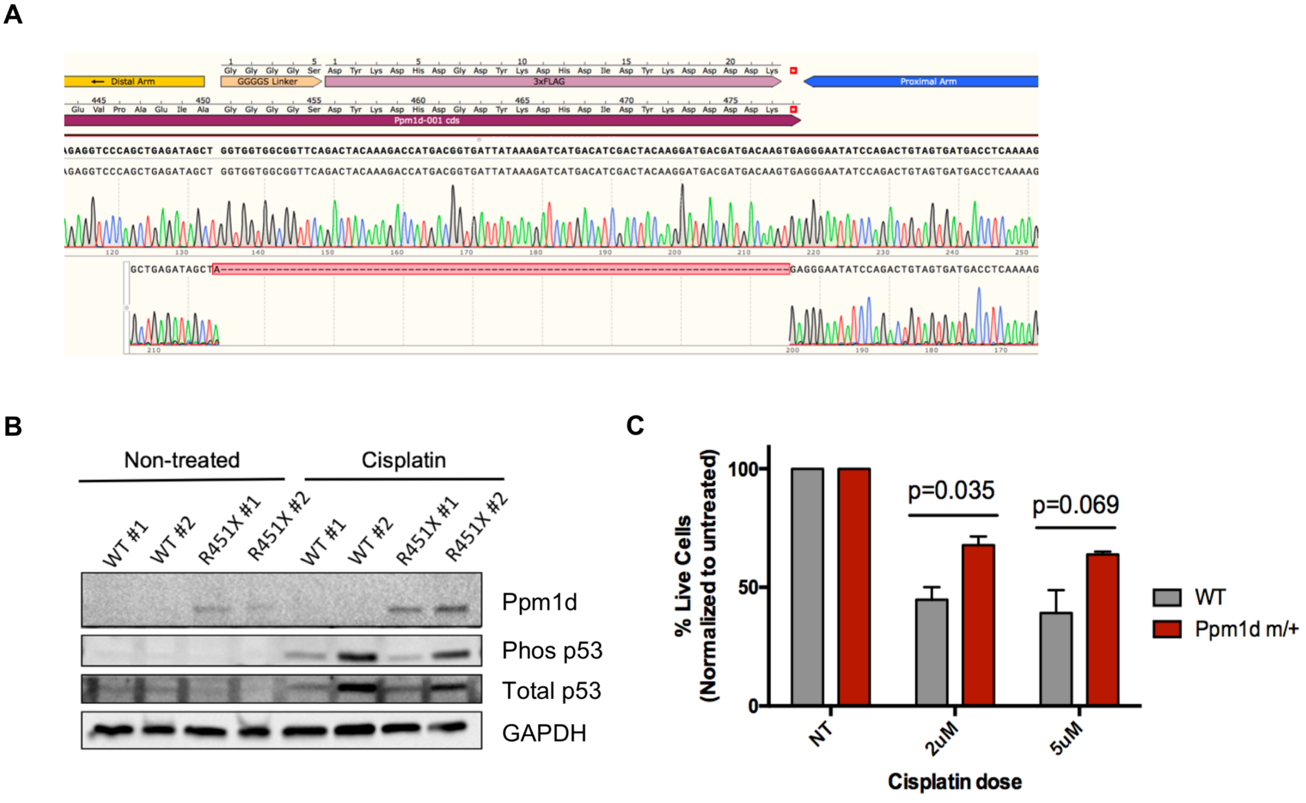

Figure S5. Characterization of the DNA damage response and apoptosis in the *Ppm1d* R451X knock-in germline mouse (Related to Figure 6). (a) Sanger sequencing trace of the mutant allele in the R451X founder mouse. **(b)** Immunoblot of the mouse embryonic fibroblast (MEF) cells generated from *Ppm1d* R451X mutant mice and their wildtype (WT) littermates, in the absence or presence of cisplatin (1uM for set #1 and 2uM for set #2). The blot was probed with the indicated antibodies. Data from two biological replicates (derived from different embryos, labeled #1 and #2) are shown for each genotype. **(c)** Analysis of apoptosis in the R451X MEFs in the absence or presence of cisplatin. Data are from two biological replicates for each genotype.

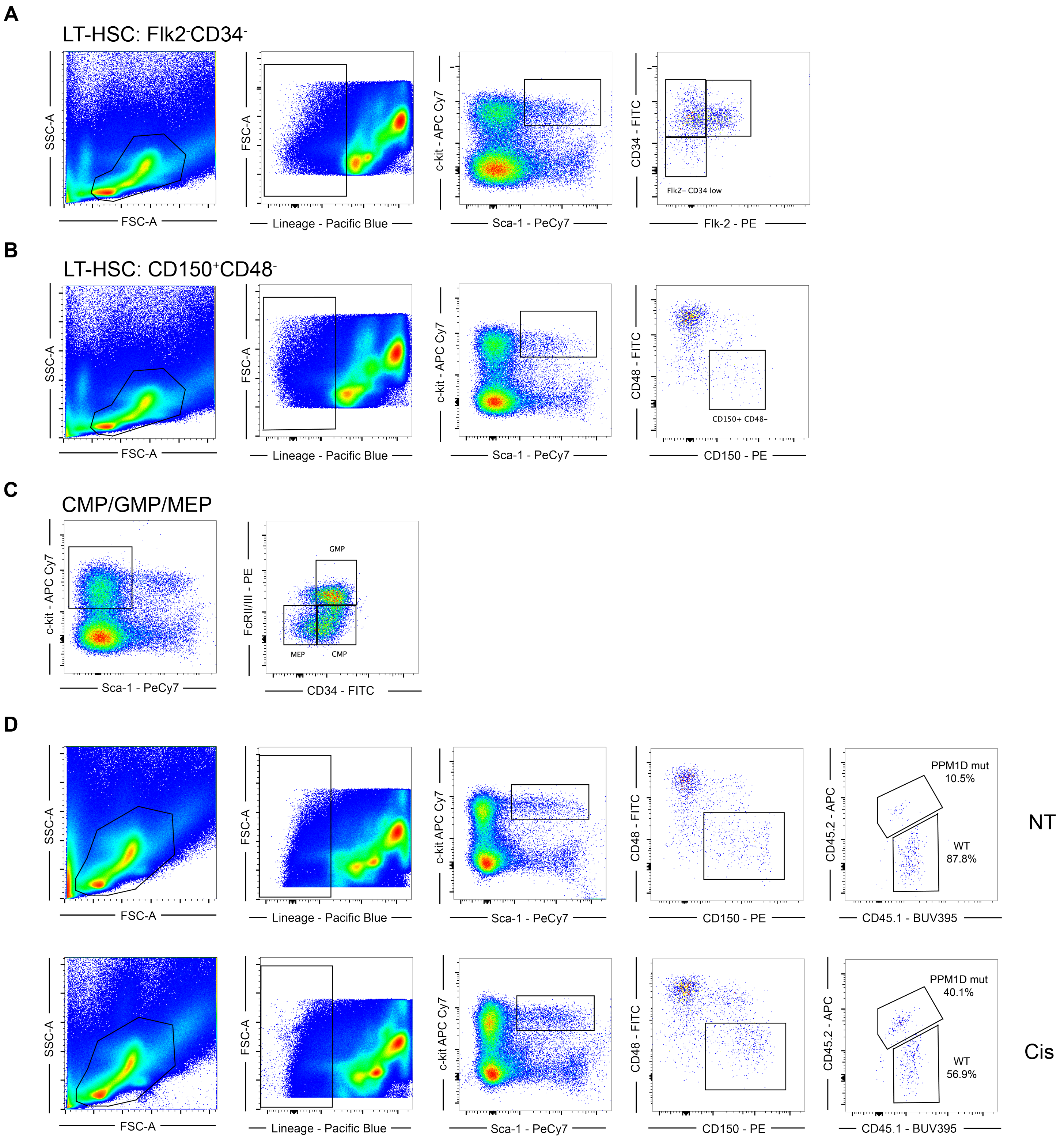

Figure S6. Flow cytometry gating scheme for HSC and progenitor populations (Related to Figure 6). (a) LT-HSC gating scheme: $cKit^{+}Lin^{-}Sca1^{+}$ $Flk2^{-}$ $CD34^{-}$. **(b)** Alternative LT-HSC gating scheme: $cKit^{+}Lin^{-}Sca1^{+}$ $CD150^{+}$ $CD48^{-}$. **(c)** CMP gating scheme: $cKit^{+}$ $Sca1^{-}$ $CD34^{+}$ FcR II/III^{-}. **(d)** Representative gating of the LT-HSC populations distinguished by the CD45 allelic markers (CD45.1 population and CD45.1/CD45.2 population) in the competitive transplants. Representative plots are shown for the untreated cohort (NT) and cisplatin-treated cohort (Cis), from the competitive bone marrow transplantation.

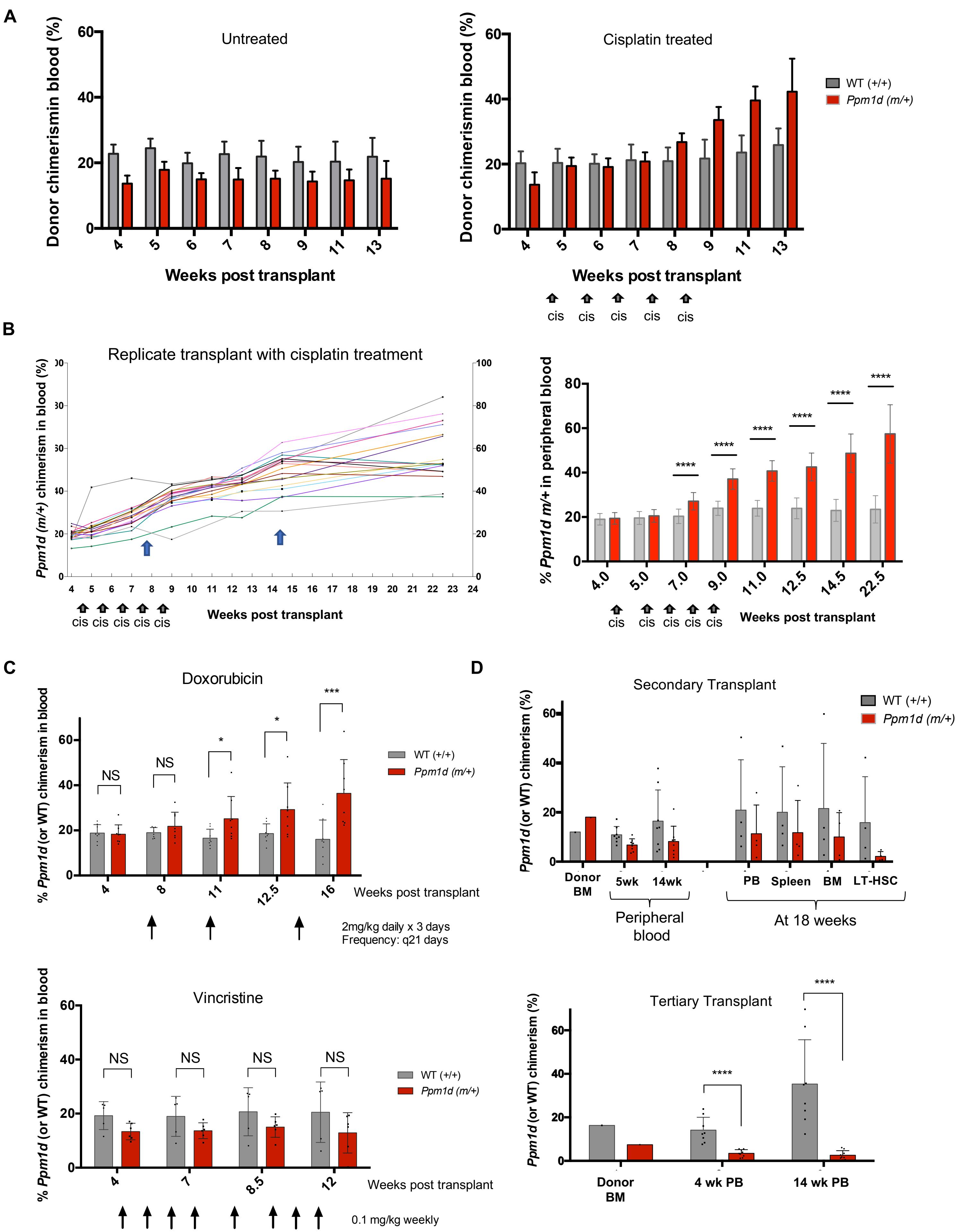
A
Untreated
Donor chimerismin blood (%)
Weeks post transplant
Cisplatin treated
Donor chimerismin blood (%)
Weeks post transplant
cis cis cis cis cis
WT (+/+)
Ppm1d (m/+)
B
Replicate transplant with cisplatin treatment
Ppm1d (m/+) chimerism in blood (%)
cis cis cis cis cis
Weeks post transplant
% Ppm1d m/+ in peripheral blood
cis cis cis cis cis
Weeks post transplant
C
Doxorubicin
% Ppm1d (or WT) chimerism in blood
NS
NS
Weeks post transplant
2mg/kg daily x 3 days
Frequency: q21 days
WT (+/+)
Ppm1d (m/+)
Vincristine
% Ppm1d (or WT) chimerism in blood
NS
NS
NS
NS
Weeks post transplant
0.1 mg/kg weekly
WT (+/+)
Ppm1d (m/+)
D
Secondary Transplant
Ppm1d (or WT) chimerism (%)
Donor BM
5wk
14wk
PB
Spleen
BM
LT-HSC
Peripheral blood
At 18 weeks
WT (+/+)
Ppm1d (m/+)
Tertiary Transplant
Ppm1d (or WT) chimerism (%)
Donor BM
4 wk PB
14 wk PB

Figure S7. Additional data from the mouse competitive bone marrow transplants (Related to Figures 6 and 7). (a) Left panel: Exact (un-normalized) peripheral blood chimerism is shown from the competitive bone marrow transplant performed in the absence of chemotherapy (corresponds to Figure 7a). Right panel: Exact peripheral blood chimerism in the competitive bone marrow transplant, with cisplatin treatment (corresponds to Figure 6d). **(b)** Left panel: A repeat competitive transplant with 20% *Ppm1d* mutant whole bone marrow cells competed against 80% WT cells was performed (n=15 per group). Following engraftment (4 weeks after transplant), the baseline peripheral blood chimerism was determined, and 5 weekly cycles of cisplatin treatment (depicted by the gray arrows) was initiated. Changes in *Ppm1d* mutant chimerism were tracked over 19 weeks (as denoted on the x-axis). Each colored line in the left graph represents an individual mouse. The blue arrows indicate two waves of *Ppm1d* mutant expansion observed at 8 weeks and 14 weeks. Right panel: An accompanying bar graph showing the average peripheral blood chimerism over time. **(c)** Competitive bone marrow transplantation with doxorubicin and vincristine treatment. Transplanted bone marrow consisted of 20% *Ppm1d*-mutant mixed with 80% WT cells or 20% WT control cells mixed with 80% WT cells. Eight weeks after initial transplant, the mice were treated with either 3 rounds of doxorubicin (each round: 2mg/kg IP x 3 days, every 21 days, n=8) or 8 injections of vincristine (0.1mg/kg IP, once weekly, n=5). The black arrows denote timing of treatment. Peripheral blood chimerism was assessed at the indicated time-points by flow cytometry. **(d)** The peripheral blood chimerism of R451X mutant cells (*Ppm1d* m/+) or wild-type control cells (*Ppm1d* +/+) in the secondary serial transplant recipients is shown at multiple time-points 5, 14, and 18 weeks after serial transplant (n=8/group). At 18 weeks, the mice were sacrificed and chimerism was assessed in the spleen, bone marrow, and LT-HSC compartments (n=4/group). A tertiary serial transplant was then performed by transplanting 3 million whole bone marrow cells from the secondary transplant recipients into lethally irradiated recipient mice. The peripheral blood chimerism of the tertiary transplant recipients is shown at 4 and 14 weeks (n=8/group). P values are indicated by *<0.05, **<0.01, ***<0.001, ****<0.0001.

Table S1. Clinical characteristics of the t-AML/t-MDS cohort (Related to Figure 1).

**Overall**		**n=156**
Median age at diagnosis, y (25th – 75th%)		67 (58-72)
Sex	Male (%)	53.8
	Female (%)	46.2
Race	White (%)	79.5
	Hispanic (%)	10.3
	Black (%)	5.8
	Other (%)	4.4
Prior condition (%)	Cancer	99.4
	Autoimmune	0.6
Prior Malignancy count (%)	1	73.7
	2	18.6
	3	7.1
Prior chemotherapy (%)		89.7
Prior XRT (%)		51.9
T-MN diagnosis category	t-AML (%)	49.4
	t-MDS (%)	50.6
Hematologic parameters, Median (25th, 75th)	BM blast	11.5 (3.25, 40.75)
	Hemaglobin	9.2 (8, 10.5)
	LDH	610 (465, 952.5)
	WBC	3.7 (1.8, 5.6)
	Platelet	47 (26, 84)

Table S2. Clinical characteristics of patients with *de novo* versus therapy-related AML/MDS (Related to Figure 1).

	De novo cohort n=228[1]	% or IQR	t-MN cohort n=156	% or IQR	p value
**Diagnosis**					
MDS	107	47%	79	51%	0.475
AML	121	53%	77	49%	
**Sex**					
Female	72	32%	72	46%	0.004
Male	156	68%	84	54%	
**Median Age**	65	57-72	67	58-72	0.536
**Median WBC**	3.85	2.0-8.2	3.7	1.8-5.5	0.167
**Median Hgb**	9.8	8.8-11.1	9.2	8.05-10.5	<0.001
**Median Plt**	62	36-134.8	47	26-83	<0.001
**Median BM blast**	16	4.3-40	11.5	3.8-40	0.451
**Cytogenetics**					
Complex karyotype	49	22%	67	44%	<0.001
Normal karyotype	90	40%	26	17%	<0.001
Inv 16 or t(8;21)	10	4.4%	3	2%	0.154
MLL rearrangement	7	3.1%	16	11%	0.003
PML-RARA	4	1.8%	0	0%	0.124
**Prior history of cancer**	44	19.6%	155	99%	<0.001
**Prior exposure to chemotherapy**	0	0%	140	90%	<0.001
**Prior exposure to radiation**	0	0%	81	52%	<0.001

Table S3. Clinical characteristics of patients with mutated *PPM1D* (Related to Figure 2).

	Overall n=156[1]	*PPM1D* n=31	Non-*PPM1D* n=125	p value
**Prior malignancy, n (%)**	155 (99.4)	30 (96.6)	125 (100)	0.199
**Prior chemo, n (%)**	140 (89.7)	30 (96.8)	110 (88.0)	0.129
**Prior XRT, n (%)**	81 (51.9)	15 (48.4)	66 (52.8)	0.66
**t-MN category**				
t-MDS, n (%)	79 (50.6)	16 (51.6)	63 (50.4)	0.904
t-AML, n (%)	77 (49.4)	15 (48.4)	62 (49.5)	
**Chromosomal changes, n (%)** [2]				
Complex	67 (44.1)	17 (54.8)	50 (41.3)	0.176
Chr 5	47 (30.9)	14 (45.2)	33 (27.3)	0.055
Chr 7	45 (29.6)	11 (35.5)	34 (28.1)	0.422
Inv 16	3 (2)	0 (0)	3 (2.5)	0.502
11q23	16 (10.5)	3 (9.7)	13 (10.7)	0.582
Normal karyotype	26 (16.7)	4 (12.9)	22 (17.6)	0.768
**Extramedullary disease, n (%)**	5 (3.2)	0 (0)	5 (4)	0.372
**Primary cancer, n (%)**				
Lymphoma				
Breast	44 (31.4)	12 (8.6)	32 (23.4)	0.059
Prostate	34 (24.3)	3 (2.1)	31 (22.2)	0.131
Myeloma	19 (13.6)	1 (0.7)	18 (12.9)	0.197
Squamous cell	11 (7.9)	2 (1.4)	9 (6.4)	1
Lung	9 (6.4)	3 (2.1)	6 (4.3)	0.202
Colon	8 (5.7)	3 (2.1)	5 (3.6)	0.152
Ovarian	7 (5.0)	0 (0)	7 (5.0)	0.352
Bladder	5 (3.6)	2 (1.4)	3 (2.1)	0.217
other	5 (3.6)	0 (0)	5 (3.6)	0.586
	22 (15.7)	2 (1.4)	20 (14.3)	0.366

[1] Percentages in each given category are based on the total number of cases with sufficient information
[2] Patients are not classified into a unique genetic subset, and can be included in more than one class

Table S4. Complete sequences of custom oligonucleotides (Related to STAR methods)

Oligonucleotide	Sequence	Note
human *PPM1D* Exon 6_30:	GGGTCCTTAGAATTCACCCT	sgRNA
human *PPM1D* Exon 6_44:	GGAAGGCATTGCTACGAACC	sgRNA
human *PPM1D* Exon 6_45:	GGGCTAAAGCCCTGACTTTA	sgRNA
human *ENAM*_60:	GGGATGATGTGTCCACGCTG	sgRNA
human *ENAM*_68:	GGGGAACTGGCTTCAGGAAA	sgRNA
mouse *Ppm1d* Exon 6 (for R451X model):	GTCCCAGCTGAGATAGCTAG	sgRNA
HDR template for R451X model:	AGAAGTTTTTAGAGGTCCCAGCT GAGATAGCTGGTGGTGGCGGTTC AGACTACAAAGACCATGACGGTG ATTATAAAGATCATGACATCGAC TACAAGGATGACGATGACAAGTG AGGGAATATCCAGACTGTAGTGA TGACCTCAAAAGACTCAGAGACA CTTGAAGAAAATTGCCCCAAAGC CCTGACTTTAAGG	Single-stranded oligo
Fwd - *PPM1D* Exon 6_30/44 deletion	TGCATAGATTTGTTGAGTTCTGG	PCR primer
Rev - *PPM1D* Exon 6_30/44 deletion	TGGTTCTGGATCTTTTGAGGGT	PCR primer
Fwd - mouse *Ppm1d* R451X genotyping	AGGCTGAGCTCTAAGGACCA	PCR primer
Rev - mouse *Ppm1d* R451X genotyping	ATTGGCTGGAGGGGTTCTTT	PCR primer
Fwd – Set 1: human *PPM1D* ex 6 amplicon sequencing	TGCATAGATTTGTTGAGTTCTGG	PCR primer
Rev – Set 1: human *PPM1D* ex 6 amplicon sequencing	AGGCCAATTGGAAGGCTATT	PCR primer
Fwd – Set 2: human *PPM1D* ex 6 amplicon sequencing	ATTGCGCTAAAGCCCTGAC	PCR primer
Rev – Set 2: human *PPM1D* ex 6 amplicon sequencing	TCTTCTGGCCCCTAAGTCTG	PCR primer
